# LIM domain only 7: a novel driver of immune evasion through regulatory T cell differentiation and chemotaxis in pancreatic ductal adenocarcinoma

**DOI:** 10.1038/s41418-024-01358-7

**Published:** 2024-08-14

**Authors:** Shangnan Dai, Yunpeng Peng, Guangfu Wang, Chongfa Chen, Qiuyang Chen, Lingdi Yin, Han Yan, Kai Zhang, Min Tu, Zipeng Lu, Jishu Wei, Qiang Li, Junli Wu, Kuirong Jiang, Yi Zhu, Yi Miao

**Affiliations:** 1 Pancreas Center, The First Affiliated Hospital of Nanjing Medical University, 300 Guangzhou Road, Nanjing, 210029 Jiangsu Province PR China; 2 Pancreas Institute, Nanjing Medical University, Nanjing, 210029 Jiangsu Province PR China; 3 Pancreas Center, The Affiliated BenQ Hospital of Nanjing Medical University, Nanjing, China

**Keywords:** Cancer microenvironment, Tumour heterogeneity, Epigenetics, Chemokines, Immune evasion

## Abstract

With advancements in genomics and immunology, immunotherapy has emerged as a revolutionary strategy for tumor treatment. However, pancreatic ductal adenocarcinoma (PDAC), an immunologically “cold” tumor, exhibits limited responsiveness to immunotherapy. This study aimed to address the urgent need to uncover PDAC’s immune microenvironment heterogeneity and identify the molecular mechanisms driving immune evasion. Using single-cell RNA sequencing datasets and spatial proteomics, we discovered LIM domain only 7 (LMO7) in PDAC cells as a previously unrecognized driver of immune evasion through Treg cell enrichment. LMO7 was positively correlated with infiltrating regulatory T cells (Tregs) and dysfunctional CD8^+^ T cells. A series of in vitro and in vivo experiments demonstrated LMO7’s significant role in promoting Treg cell differentiation and chemotaxis while inhibiting CD8^+^ T cells and natural killer cell cytotoxicity. Mechanistically, LMO7, through its LIM domain, directly bound and promoted the ubiquitination and degradation of Foxp1. Foxp1 negatively regulated transforming growth factor-beta (TGF-β) and C-C motif chemokine ligand 5 (CCL5) expression by binding to sites 2 and I/III, respectively. Elevated TGF-β and CCL5 levels contribute to Treg cell enrichment, inducing immune evasion in PDAC. Combined treatment with TGF-β/CCL5 antibodies, along with LMO7 inhibition, effectively reversed immune evasion in PDAC, activated the immune response, and prolonged mouse survival. Therefore, this study identified LMO7 as a novel facilitator in driving immune evasion by promoting Treg cell enrichment and inhibiting cytotoxic effector functions. Targeting the LMO7-Foxp1-TGF-β/CCL5 axis holds promise as a therapeutic strategy for PDAC.

**Figure:**
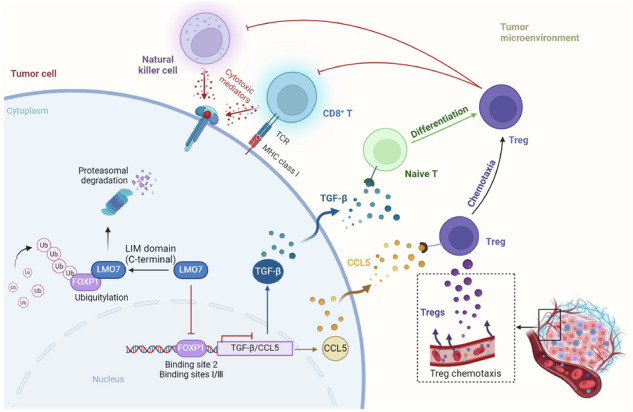
**Graphical abstract** revealing LMO7 as a novel facilitator in driving immune evasion by promoting Tregs differentiation and chemotaxis, inducing CD8^+^ T/natural killer cells inhibition.

**Graphical abstract** revealing LMO7 as a novel facilitator in driving immune evasion by promoting Tregs differentiation and chemotaxis, inducing CD8^+^ T/natural killer cells inhibition.

## Introduction

Pancreatic ductal adenocarcinoma (PDAC) is an exceptionally malignant and aggressive tumor with a 5-year survival rate of 12% [1]. Surgery is the primary therapeutic approach for PDAC. Unfortunately, over 80% of individuals with PDAC encounter a recurrence of the disease following surgical resection [2]. Furthermore, chemotherapy, as an adjuvant treatment, can only provide limited survival benefits or transient disease stabilization [3, 4].

Recently, remarkable strides have been made for numerous cancer types with advances in immunotherapy; however, their effectiveness is limited to a small subset of PDAC cases [5–7]. PDAC demonstrates an immunologically “cold” tumor microenvironment (TME), primarily characterized by substantial infiltration of immune cells that promote tumors, such as regulatory T cells (Tregs) [8–10]. Tregs, a subset of immunosuppressive cells, are crucial for preserving self-tolerance and immunological balance [11]. In the context of tumor immunity, Tregs hinder immune surveillance against cancer in healthy individuals and impede the antitumor immune response in those with tumors [12, 13]. Consequently, Tregs contribute to the acceleration of immune evasion by tumor cells, thereby promoting the development and progression of tumors across different cancer types [14–17] .Activation and accumulation of Tregs in the TME have been demonstrated in various tumor types, such as lung adenocarcinoma, glioblastoma, clear cell renal carcinoma, bladder, prostate, liver, and breast cancers, and PDAC[10, 18–22].

Tregs foster the proliferation of tumor cells by inhibiting antigen-presenting cells (APCs), depleting a crucial cytokine necessary for the activation and function of effector T cells and generating immunosuppressive factors [23, 24], which leads to the formation of an immunosuppressive TME. Simultaneously, tumor cell cytokines promote Treg cell proliferation. The resulting chemotactic factor gradient can recruit Treg cells to the tumor. Transforming growth factor-beta (TGF-β) and interleukin 10 (IL10) have been shown to induce the differentiation and expansion of Treg cells, thereby upregulating the expression of forkhead box P3 transcription factor (FOXP3) and cytotoxic T lymphocyte antigen 4 (CTLA-4) [13, 25, 26]. TGF-β from mouse melanoma and breast cancer cells promotes Treg cell proliferation [27]. B Bintrafusp alfa is a bifunctional fusion protein capable of blocking programmed death ligand 1 (PD-L1) and neutralizing TGF-β. It has been demonstrated in a clinical trial for human papillomavirus-unrelated head and neck squamous cell carcinoma that bintrafusp alfa can reverse the immune suppression of Tregs in tumors [28]. There has been an enduring interest in targeting chemokines for cancer treatment. Recently, it has been demonstrated that CD73 upregulated CCL5 via the p38-STAT1 pathway, recruiting Tregs to tumors and creating an immunosuppressive microenvironment [9]. Given their pivotal roles, Tregs are regarded as a crucial therapeutic target in cancer immunotherapy. However, the heterogeneity of PDAC poses challenges to relevant clinical trials, and the achieved outcomes have not been universally satisfactory. Therefore, it is necessary to unravel the immune microenvironment heterogeneity in PDAC and focus on targeting cytokines. In this study, we discovered the association of LIM domain only 7 (LMO7) in tumor cells with the enrichment of Tregs and immune evasion at the single-cell level.

A member of the LIM protein family, LMO7, is expressed in various tissues and organs, including myocardial and muscle cells. LMO7 plays a crucial role in processes, such as cell differentiation, migration, adhesion, and polarity [29–32]. For instance, by interacting with the nuclear membrane protein emerin, LMO7 regulates the transcription of emerin and numerous other genes associated with muscle, such as CREBBP, NAP1L1, LAP2, and RBL2 [33]. Additionally, LMO7 is implicated in various diseases, such as muscle atrophy, heart disease, and hearing impairment [34–36]. Recently, it has been reported that LMO7 can promote the proliferation and metastasis of breast, prostate, and thyroid cancers [37–39]. However, contrasting results have been observed in lung cancer [40, 41]. Further research is needed to confirm the role of LMO7 in tumors. Our preliminary studies have identified high expression of LMO7 in PDAC [42]. Recently, the immune regulatory role of LMO7 has been found in preliminary studies. LMO7 modulates the tumor immune microenvironment (TIME) [43] and regulates dendritic cells (DCs) and CD8^+^ T cells intrinsically through the STING pathway [44]. However, these studies are limited to bioinformatics analysis or have not elucidated the mechanism by which LMO7 inhibits CD8^+^ T cells and immune response in PADC. The function of LMO7 in regulating the anti-tumor immune response of PDAC cells and immune molecules remains largely unknown.

In this study, our objective is to identify the immune microenvironment heterogeneity in PDAC using single-cell RNA sequencing and spatial proteomics, aiming to elucidate the role of tumor cell LMO7 in immune evasion. We seek to unveil, for the first time, the specific mechanism by which LMO7 influences immune cells in tumors. Furthermore, we aim to assess the feasibility of therapeutic strategies targeting this mechanism in PDAC. This study may provide novel insights into determining precise immunotherapeutic strategies for patients with specific PDAC genotypes and phenotypes.

## Materials and methods

### Tissue samples

Seventy pairs of tumor tissue and adjacent tissue were collected from PDAC patients at the Pancreas Center of the First Affiliated Hospital of Nanjing Medical University. These patients underwent radical surgical resection at the Pancreas Center of the First Affiliated Hospital of Nanjing Medical University between 2009 and 2016. Pathological confirmation was performed for pancreatic ductal epithelial cell carcinoma, and their overall survival time was regularly recorded through follow-up visits. The tissue microarrays (TMA) HPanA120Su02 were obtained from Shanghai Outdo Biotech (Shanghai, China). All participants provided informed consent. All human tissue research in this study had the approval of ethics committees of the First Affiliated Hospital of Nanjing Medical University (Nanjing, China).

### Animal experiments

Animal experiments were conducted following the NIH guidelines and protocols, approved by the Institutional Animal Care and Use Committee (IACUC) of Nanjing Medical University. Female C57BL/6 mice were purchased from Charles River (Beijing, China), and NCG mice were purchased from GemPharmatech (Nanjing, China). Mice were maintained under specific pathogen-free (SPF) conditions with a 12-hour light/dark cycle (150–300 lux), environmental temperature of 20–26 °C, humidity ranging from 40 to 70%, and ventilation occurring four times per hour. Panc02 cell suspension (approximately 1 × 10^6^ cells) mixed with Matrigel (BD Biosciences) at a 1:1 ratio was prepared as a 50 μL injection. The mice were randomly divided into groups. Under sterile conditions, mice were anesthetized and secured on a surgical table, and the left abdominal skin was disinfected with iodine. A small incision was made in the left abdominal wall. The pancreas was carefully exposed, and the cell suspension was slowly injected into the tail of the pancreas using a disposable syringe to prevent bleeding or leakage. The injection site was covered with tissue adhesive (Vetbond, 3 M), and the pancreas was returned to its original position. The abdominal wall and skin were sutured, and pain relief and antibiotics were administered to prevent infection. Mouse recovery was monitored, with necessary treatments or sacrifice performed as needed. Tumors were ultimately collected for analysis using flow cytometry and pathological examinations. Data are presented as mean ± standard error of mean (SEM). *n* = 6 mice per group. Statistical significance was determined using two-tailed unpaired *t*-test.

### Cell culture

All cell lines used in this experiment, including CFPAC-1 and Mia PaCa-2, were obtained from the National Collection of Authenticated Cell Cultures. Panc02 was obtained from the Cell Resource Center, Peking Union Medical College (part of the National Science and Technology Infrastructure, the National Biomedical Cell-Line Resource, NSTI-BMCR). All cells were cultured in dishes (NEST Biotechnology, China) with Dulbecco’s modified Eagle’s medium (DMEM; 319-005-CL, Wisent, Canada) supplemented with 10% fetal bovine serum (FBS; 920040, Wisent, Canada), penicillin (100 U/mL), and streptomycin (100 μg/mL) (C0222, Beyotime, China). Cells were cultured in a humidified incubator at 37 °C with 5% CO_2_. Cell lines were authenticated and checked for mycoplasma using short tandem repeat DNA profiling.

### Plasmids and vectors

LMO7 small interfering RNAs (siRNAs) were constructed by Ribobio (Guangzhou, China). The LMO7, Foxp1 overexpression, shLMO7, and shFoxp1 lentiviral vectors were purchased from Genechem (Shanghai, China). All plasmids with site deletions and mutations were synthesized by Genechem (Shanghai, China).

### Flow cytometry

Following the manufacturer’s instructions, the cells were incubated with the appropriate antibodies at room temperature for 30 min. Subsequently, membrane permeabilization was performed for intracellular and nuclear antibodies, followed by another round of antibody incubation. After washing the samples twice with PBS, they were analyzed using a flow cytometer. The gating strategies employed for flow cytometry staining are presented in the Supplementary Figures. The following antibodies were used: CD45 (103106, BioLegend, USA), CD25 (102043, BioLegend), FOXP3 (320014, BioLegend), CD3 (100220, BioLegend), CD4 (100539, BioLegend), CD8a (100706, BioLegend), NK 1.1 (108710, BioLegend), IFN-γ (505830, BioLegend), Compensation Beads (424602, BioLegend), Zombie Aqua Fixable Viability Kit (423101, BioLegend), and Foxp3/Transcription Factor Staining Buffer (00-5523-00, ThermoFisher).

### Peripheral blood mononuclear cell isolation

Initially, the syringe walls were pre-wetted with heparin, and blood was collected immediately. The blood samples were then diluted at a 1:1 ratio with an appropriate culture medium or PBS + 2% FBS. Ficoll was added to a new tube, and the diluted blood was gently layered on top of the density gradient medium without mixing the two layers. After centrifugation at 800 × *g* for 20–30 minutes with the brake off, the cell layer containing mononuclear cells was carefully collected, washed twice with an appropriate buffer, and, if necessary, treated with ACK lysis buffer to remove residual red blood cells before additional washing. The cells were then ready for downstream applications, followed by cell counting.

### Isolation of naive CD4^+^ T cells

Peripheral blood from healthy individuals was collected, and PBMCs were isolated using Ficoll. Subsequently, Naive T cells were obtained using the Milteniyi Naive CD4^+^ T Cell Isolation Kit (130-094-131, Miltenyi Biotec, Germany). Naive CD4^+^ T cells were isolated using a negative selection magnetic bead sorting method, targeting a range of antigens (including CD8, CD14, CD15, CD16, CD19, CD25, CD34, CD36, CD45RO, CD56, CD123, TCRγ/δ, HLA-DR, and CD235a). After treatment with specific antibody cocktails and magnetic separation, the enriched and purified naive CD4^+^ T cells were collected for further analysis, including flow cytometry, to assess purity.

### Isolation of Tregs

Regulatory T cells were isolated using a combination of negative and positive selection magnetic bead sorting (130-094-125, Miltenyi Biotec, Germany). The process involved a mixture of biotinylated monoclonal antibodies against various antigens (CD8, CD14, CD15, CD16, CD19, CD36, CD56, CD123, TCRγ/δ, CD235a) and CD25. The steps included preparation of the sample, magnetic labeling of non-CD4^+^ cells, magnetic separation of non-CD4^+^ cells, magnetic labeling of Tregs, and a second round of magnetic separation for enhanced purity. Flow cytometry was performed for purity analysis.

### Mouse single-cell suspension preparation

For the preparation of mouse single-cell suspension for experimental procedures, fresh tumor tissues obtained from mice were meticulously processed. The tissues were initially cut into small fragments and subjected to a combination of mechanical and enzymatic dissociation using specific buffers and enzymes according to kit instructions (130-096-730, Miltenyi Biotec, Germany). The resulting cell mixture was then filtered through a 70 μm strainer to eliminate residual tissue debris, followed by centrifugation and washing to obtain a purified single-cell suspension.

### Co-culture experiment

Approximately 2 × 10^6^ naive T cells were initially added to each well, and a 1:1 ratio of CD3/CD28 beads and 100 U/mL rIL-2 was simultaneously introduced for a 48-hour cultivation period. Following this, the cells were directly co-cultured with PDAC cells or seeded into shared chambers for indirect co-culture. The co-culture lasted for 5 days, after which the T cells were collected, washed, filtered, and subjected to flow cytometry analysis to assess the outcomes of the interaction.

### T-cell chemotaxis assay

In the chemotaxis experiment, Tregs or PBMCs were placed in the upper chamber of a co-culture well, whereas PDAC cells were placed in the lower chamber. After 24 hours of co-culture, cells from the lower chamber were collected, and the quantity of Tregs was analyzed using flow cytometry.

### EDU assay

T cells were cultured in the presence of EDU, a thymidine analog that be incorporated into actively synthesizing DNA. After a specified incubation period, the cells were washed, fixed, and subjected to a click reaction, enabling the covalent binding of a fluorescent dye to the incorporated EDU. Flow cytometry was then employed to analyze the percentage of cells with EDU incorporation, providing insights into the actively proliferating cell population.

### ChIP assays

The assays were performed using a ChIP Assay Kit (P2078, Beyotime, Shanghai, China). CFPAC-1 cells were fixed with 1% formaldehyde and cross-linking was terminated using 0.125 M glycine. After collecting and lysing cells, chromatin fragments were obtained through sonication using a VCX750 at 25% power for 4.5 s pulses with 9 s intervals, repeated 14 times. Samples were pre-cleared with Protein A/G Agarose for 30 minutes at 4 °C. Following the extraction of the 1% input sample, the remaining samples were evenly divided and subjected to incubation with an anti-Foxp1 antibody or IgG conjugated to Protein A/G Agarose. This incubation occurred at 4 °C overnight. The antibody-protein-DNA complexes was subsequently washed with low salt, high salt, LiCl, and TE buffers to remove nonspecific binding. The complexes were eluted using 250 μl elution buffer (10% SDS and 1 M NaHCO3), followed by de-crosslinking and RNaseA and proteinase K treatment. The DNA fragments were purified using an omega gel extraction kit. The eluted DNA was subjected to qPCR analysis to examine specific gene or promoter regions within the DNA fragments.

### Western blotting

The cellular proteins were extracted using RIPA lysis buffer. Protein concentration was determined using the BCA assay, and the concentration was adjusted to 4 mg/mL. Samples were prepared with SDS sample buffer, boiled, and loaded for SDS-PAGE. The gel was transferred to PVDF membranes, followed by blocking, incubation with primary and secondary antibodies, and detection using chemiluminescence or colorimetric methods. The resulting chemiluminescent signals were captured for protein expression analysis. Uncropped western blots are provided in Supplementary Material.

### Quantitative real-time PCR (qRT-PCR)

Total RNA was extracted from PDAC cells using TRIzol reagent (15596026, Invitrogen, USA). The quality and concentration of RNA were assessed using Nanodrop 2000. The concentration of RNA was adjusted to 500 ng/μL. Subsequently, cDNA was synthesized through reverse transcription using the PrimeScript RT Master Mix (RR036B, TAKARA Bio, Japan). The real-time fluorescence quantitative PCR reaction was then set up with specific primers and ChamQ SYBR qPCR Master Mix (Q341-02/03 Vazyme biotech, Nanjing, China), and the reaction conditions involved an initial denaturation step, 40 cycles of denaturation, annealing, and extension, and a melting curve analysis. The relative expression of the target gene was calculated using the 2^–△△Ct^ method, with each experiment being repeated three times.

### Luciferase reporter assay

The assay was conducted following the manufacturer’s guidelines. Briefly, PDAC cells were cultured in 24-well plates until reaching 70% confluence. Subsequently, they were transfected with 1 µg of either truncated or mutated TGFβ/CCL5 promoter luciferase reporter in the specified cells, along with 0.025 μg pRL-TK for normalization. After 48 hours of transfection, cell lysates were collected, and luciferase activity was quantified using the Luciferase Assay System from Promega following the manfacturer’s instructions.

### Immunohistochemistry (IHC) and immunofluorescence

The IHC samples were sourced from the Pancreatic Center of the First Affiliated Hospital of Nanjing Medical University, and the tissue microarrays (TMA) used for multiplex immunohistochemistry (mIHC) were produced by OUTDO Biotech Co., Ltd (Shanghai, China).

The slices underwent deparaffinization and rehydration through successive treatments with xylene and graded ethanol. Heat-induced epitope retrieval (HIER) was then performed. Subsequently, slides were exposed to an anti-human primary antibody, followed by blocking endogenous tissue peroxidase using 0.3% hydrogen peroxide. Finally, a polymeric HRP-conjugated secondary antibody was applied. The immunofluorescent signal was enhanced using the Think color-7 fluorescent mIHC kit (FreeThinking Biosciences, Nanjing, China) and the fluorophore-conjugated Tyramide Signal Amplification kit (TSA; FreeThinking Biosciences) at a 1:100 dilution (staining order: 690, 480, 520, 570, 620, 780). Following Think TSA signal deposition, HIER was performed to remove tissue-bound primary/secondary antibody complexes while preserving the tyrosine-residue-bound Think-TSA signal. This process was iterated until all six markers of interest were labeled, concluding with a nuclear DAPI counterstain.

Fluorescence images were captured using a fluorescence scanner (VectraPolaris, Akoya Bio, Mass, USA), and quantitative analysis of the scanned results was conducted using the HALO Highplex FL analysis module of the pathology image analysis software HALO (version 3.4.2986.257, Indica Labs, Albuquerque, NM, USA). A computational algorithm, taking into account both the percentage of positively stained cells and the fluorescence intensity (classified as low, medium, and high), was employed. This approach yielded an H-score, a semi-quantitative measure of biomarker intensity. The analysis involved quantifying the total number of positive cells in each staining channel, categorizing them into weak, moderate, and strong intensity levels. This assessment included recording the presence of positive cells in the nucleus, cytoplasm, and membrane. Subsequently, percentages of positive cells relative to the total cell population were calculated, and the H-scores were determined for each channel. Proximity and nearest neighbor analysis were utilized to evaluate the spatial relationship between immune and LMO7^+^ PDAC cells. The following antibodies were used: CD3 (ab16669, Abcam, 1:2000), CD8 (85336, CST, 1:1200), FOXP3 (98377, CST, 1:800), LMO7 (HPA020923, Sigma, 1:300), CD163 (ab182422, Abcam, 1:2000), and GZMB (ab255598, Abcam, 1:3000).

### Co-IP assay and IP with mass spectrometry

Cells were initially harvested and subjected to centrifugation. The collected cells were then resuspended in IP lysis buffer or RIPA buffer. After centrifugation to obtain the cell lysate, the protein concentration was determined, and the lysate was divided into aliquots. Target-specific antibodies were added to each aliquot and allowed to incubate overnight at 4 °C. Protein A/G agarose beads were subsequently introduced, followed by gentle shaking to facilitate binding. The beads were then collected and washed, and the bound proteins were released by heating in SDS-PAGE sample buffer. Finally, the samples underwent SDS-PAGE analysis, and the interaction between proteins was assessed using western blotting. Mass spectrometry was performed to analyze the extracted immunoprecipitants.

### Bioinformatics analysis

Differential gene expression levels of LMO7 between normal and PDAC tissue were collected from Gene Expression Profiling Interactive Analysis 2 (GEPIA2) database (http://gepia2.cancer-pku.cn/) (*p*-value cutoff was 0.01, match TCGA normal and GTEx data). Overall Survival (OS) for LMO7 in PDAC were generated by Kaplan-Meier Plotter using GEPIA2 (group cutoff was Median). The Gene Set Cancer Analysis (GSCA) database (https://guolab.wchscu.cn/GSCA/#/immune) was used for correlation between gene expression and immune infiltration by selecting Spearman analysis methodology. Immunogenomic analysis was performed by the ImmuCellAI algorithm with 24 immunes cells.

The single-cell sequencing data (CRA001160, GSA: https://bigd.big.ac.cn/gsa) was employed to comprehensively analyze the compositional differences and dynamic changes in the TME during the malignant progression of PDAC. Gene-cell matrixes were filtered to remove cells (<200 transcripts/cell, >15% mitochondria genes). Then the matrix was imported into the R package Seurat (v 3.1.2) for subsequent analysis. The gene expression levels were normalized so that the number of unique molecular identifiers in each cell (UMI/cell) is equal to the median UMI and then natural-log transformed. Total 2000 highly variable genes were generated for performing PCA reduction dimension. Significant principle components were determined using Jackstraw. Finally, single cell clustering was visualized by UMAP (Uniform Manifold Approximation and Projection). R package (Monocle 2) was applied to conduct cellular trajectory analysis with the assumption that one-dimensional ‘time’ can describe the high-dimensional expression values, so called pseudotime analysis of single cells. The clusters identified as T cells were loaded into R environment. And we presented cell trajectory and position with tree structure in two-dimension space after log normalization and reduction dimension. Then we set the pattern of each cell in the plot according to specific markers expression level.

To facilitate a systematic analysis of cell-cell communication molecules, we utilized cell communication analysis employing both CellPhoneDB and CellChat, which serve as public repositories containing information on ligands, receptors, and their interactions.

For CellPhoneDB analysis, the membrane, secreted, and peripheral proteins within clusters from different time points were annotated. This process entails aggregating cells with analogous annotations into discrete cell states. Subsequently, interactions between these states are scrutinized, relying on the expression of receptors in one state and ligands in another. For every gene within a cluster, the proportion of cells expressing it and its mean expression are calculated. Through evaluation of ligand and receptor expression levels within each state and the application of empirical shuffling, notable ligand-receptor pairs specific to cell states are discerned.

In CellChat analysis, gene expression data from cells along with their assigned cell types were employed. The process first identified overexpressed ligands or receptors within specific cell groups. Subsequently, the gene expression data were mapped onto a protein-protein interaction network. Overexpressed ligand-receptor (L-R) interactions were recognized whenever either the ligand or receptor was found to be overexpressed. Byassigning a probability value to each interaction and conducting a permutation test, the biologically significant cell-cell communication was inferred. Finally, the communication networks were visualized using circle plots.

### Statistical analysis

All statistical analyses were conducted using SPSS Statistics version 22.0 (Chicago, Ill) and GraphPad Prism 9 (GraphPad Software, Inc., La Jolla, CA, USA). Group differences in both independent and paired samples were assessed using the *t*-test. Data were presented as the mean ± SEM (*n* = 3 independent biological replicates). One-way analysis of variance (ANOVA) with Tukey’s post hoc test was employed for comparisons among multiple groups, while two-way ANOVA was used when there were two experimental factors. Kaplan–Meier survival plots were generated, and survival analysis was conducted using log-rank tests. A *p*-value less than 0.05 was considered statistically significant.

## Results

### LMO7 promoted the formation of the immunosuppressive microenvironment and predicted poor prognosis in PDAC tumor

**Fig. 1:**
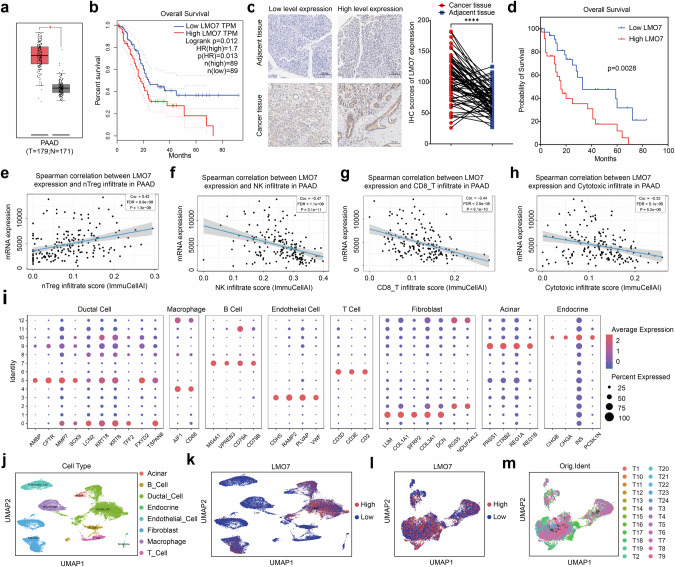
LMO7 predicts the poor prognosis that accompanies immune suppression in PDAC. **a** Expression levels of LMO7 mRNA in PAAD from TCGA and GTEx datasets. **b** Overall survival curves for low and high LMO7 mRNA expression groups. Statistical significance was determined using log-rank test. **c** Protein levels of LMO7 in PDAC tissue and adjacent non-cancerous tissue. Statistical significance was determined using two-tailed paired *t*-test. **d** Overall survival curves for LMO7 protein expression in PDAC. Statistical significance was determined using log-rank test. **e**–**h** Correlation analysis of LMO7 with immune cell infiltrations in TCGA-PAAD database. Correlation with Treg infiltration (**e**); Correlation with NK cell infiltration (**f**); Correlation with CD8^+^ T cell infiltration (**g**); Correlation with cytotoxic cell infiltration score (**h**). **i** Dot plot showing the expression level of specific marker genes for each cell type. **j** UMAP plots depicting major cell types in PDAC based on single-cell sequencing data (CRA001160), including acinar cells, B cells, ductal cells, endocrine cells, endothelial cells, fibroblasts, macrophages, and T cells. **k** UMAP plots displaying LMO7 expression of diverse cell types. **l** UMAP plots showing the classification of ductal cells based on high and low LMO7 expression. **m** UMAP plots showing the transcription heterogeneity of ductal cells colored by sample types. *p < 0.05; **p < 0.01; ***p < 0.001, ****p < 0.0001.

The analysis of pancreatic tissues from TCGA and GTEx indicated that the expression of LMO7 was significantly elevated in PDAC tissues (Fig. 1a) compared to that in normal pancreatic tissues. LMO7 was associated with a significant reduction in the overall survival of patients with PDAC (Fig. 1b). We assessed the LMO7 protein level in 70 pairs of PDAC and matched adjacent tissues through immunohistochemistry (IHC). LMO7 expression level was significantly higher in PDAC tissues than in adjacent ones (Fig. 1c). Additionally, high expression of the LMO7 protein was associated with a significant reduction in the overall survival of patients with PDAC (Fig. 1d).

To explore the relationship between LMO7 and the PDAC TME, we utilized the TCGA database to analyze the correlation between LMO7 and infiltrating immune cells in PDAC. We observed a positive correlation between LMO7 and Treg cell infiltration scores (Fig. 1e) and a negative correlation with NK cell (Fig. 1f), CD8^+^ T cell (Fig. 1g), and cytotoxic infiltration scores (Fig. 1h). These findings indicate that LMO7 expression is associated with levels of immune cell infiltration in PDAC.

Furthermore, we employed single-cell sequencing data (CRA001160, GSA: https://bigd.big.ac.cn/gsa) to comprehensively analyze the compositional differences and dynamic changes in the TME during the malignant progression of PDAC. Differential gene expression analysis was performed to generate cluster-specific marker genes, thereby defining the identity of each cell cluster (Fig. 1i). The characteristic genes for each cell cluster were consistent with well-established cell markers. For example, the expression of KRT (keratin) and PRSS1 were identified as markers for ductal and acinar cells, respectively. After gene selection, normalization, and principal component analysis, we identified eight distinct clusters, including acinar cells, B cells, ductal cells, endocrine cells, endothelial cells, fibroblasts, macrophages, and T cells (Fig. 1j). The composition of these clusters varied among samples from different sources (Fig. S1a). This indicated that alterations in cellular components occurred during the malignant progression of PDAC, revealing obvious heterogeneity among the samples.

The expression of LMO7 was plotted on a UMAP to identify the cells enriched with LMO7. The results indicated that LMO7 was predominantly expressed in ductal cells (Fig. 1k). However, our observation of LMO7 expression in fibroblast cells is also an important finding that warrants further investigation. Furthermore, we focused on ductal cells, with the color gradient from navy blue to yellow indicating a low-to-high expression of LMO7 in these cells (Fig. S1b). Moreover, ductal cells were divided into high and low LMO7 groups (Fig. 1l), and the sample sources of ductal cells with differential LMO7 expression were identified (Fig. 1m).

We further explored the differences in T-cell components between the high and low LMO7 expression groups. The results indicated that the T-cell components in the high and low LMO7 expression groups were generally consistent (Fig. S1c, e). However, based on the analysis of sample sources, the high LMO7 expression group exhibited a more heterogeneous T-cell infiltration with a broader presence of Tregs (Fig. S1d). Conversely, the samples with low LMO7 expression showed a more widespread infiltration of naive T cells, and Treg cells were limited to individual samples (Fig. S1f). Additionally, the group with high LMO7 expression had a higher proportion of Treg cell infiltration (Fig. S1g). This suggests that changes in LMO7 could be associated with the differentiation of T cells, indicating a dynamic and evolving process. Furthermore, pseudotime analysis revealed that in samples with high LMO7 expression, naive T cells showed a more pronounced Treg cell differentiation than in samples with low LMO7 expression (Fig. S1h–k). This trend raises the possibility that elevated LMO7 expression may be associated with the differentiation of Tregs.

### Multiplex immunohistochemistry revealed the spatial distance relationship between immune and LMO7+ cells in PDAC

**Fig. 2:**
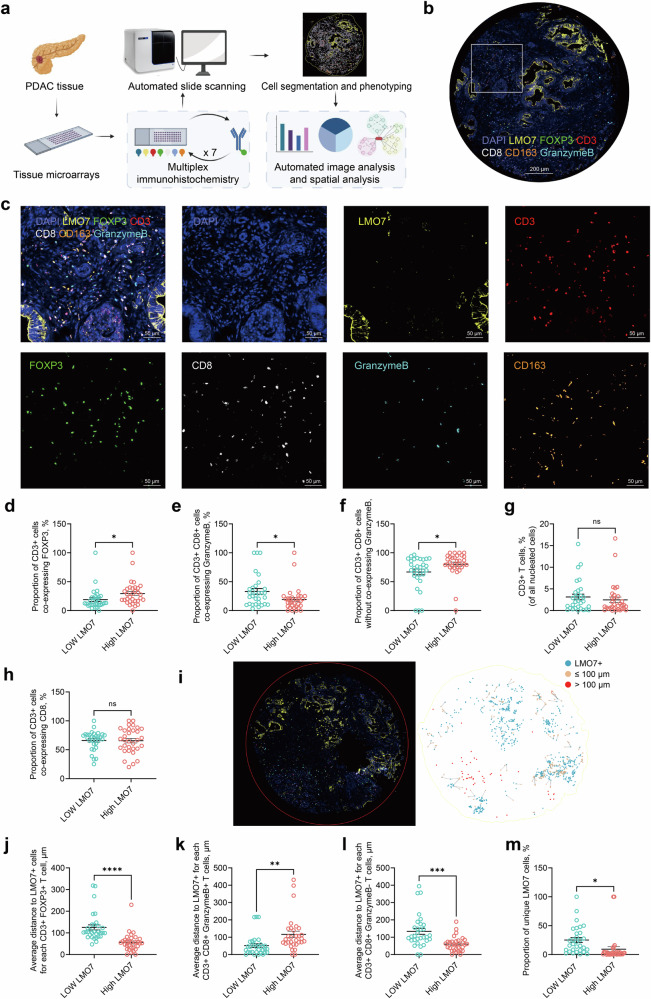
Distance between suppressive immune cells and LMO7^+^ cells decreased in PDAC. **a** The schematic representation illustrating the experimental design and analytical methods employed in this study. **b** The mIHC panel for PDAC tissues, incorporating LMO7 and immunological markers. **c** Representative images depicting staining with antibodies against the indicated marker. **d** Proportion of CD3^+^ lymphocytes co-expressing FOXP3. **e** Proportion of CD3^+^ CD8^+^ lymphocytes co-expressing GranzymeB. **f** Proportion of CD3^+^ CD8^+^ lymphocytes without co-expressing GranzymeB. **g** CD3^+^ T cells percentage of all nucleated cells. **h** Proportion of CD3^+^ lymphocytes co-expressing CD8. **i** Halo spatial analysis plot illustrating LMO7^+^ cells (blue), immune cells within 100 μm (orange), and those >100 μm away (red) from an LMO7^+^ cell. **j** Average distance to LMO7^+^ cells for each CD3^+^ FOXP3^+^ T cell. **k** Average distance to LMO7^+^ cells for each CD3^+^ CD8^+^ GranzymeB^+^ T cells. **l** Average distance to LMO7^+^ cells for each CD3^+^ CD8^+^ GranzymeB^-^ T cells. **m** Proportion of unique LMO7 cells. Data are presented as mean ± SEM. The *p*-value was determined using unpaired two-sided Student’s *t*-test. *p < 0.05; **p < 0.01; ***p < 0.001; ****p < 0.0001.

To further investigate LMO7’s impact on tumor immune responses and understand immune microenvironment heterogeneity in PDAC, we conducted single-cell spatial proteomic analysis using mIHC on a tissue microarray comprising 66 PDAC and 54 adjacent tissue specimens (Fig. 2a). We developed a 7-marker panel for PDAC tissues, including LMO7 and immunological markers (CD3, FOXP3, CD8, GranzymeB, and CD163) using the Halo® image analysis platform (Fig. 2b). Six subsets of immune cells were identified, comprising CD3^+^, CD3^+^ CD8^+^, and CD3^+^ FOXP3^+^ T cells; CD3^+^ CD8^+^ GranzymeB^+^ and CD3^+^ CD8^+^ GranzymeB^-^ cells, and CD163^+^ macrophages (Fig. 2c). The scanned images underwent cell segmentation, and the expression levels of markers in each cell within the images were quantified. Excluding three sections of detached tissues, PDAC tissues were stratified into high- and low-expression groups based on LMO7 expression (Table 1).

**Table 1:** Summary of mIHC results.

Characteristics	High LMO7 (32)	Low LMO7 (31)	*p*-value
Total number of nucleated cells assessed	4834.66 ± 429.23	5733.39 ± 469.39	0.1690
LMO7+ cells	838.22 ± 135.60	98.39 ± 13.59	0.0000*
LMO7+ cells, %	16.46 ± 12.12	1.75 ± 1.10	0.0000*
CD3, CD8, FOXP3 expression			
CD3+ T cells, % (as % of all nucleated cells)	2.48 ± 3.57	3.15 ± 3.53	0.4625
CD3+ CD8+ T cells, % (as % of all nucleated cells)	1.82 ± 3.18	2.24 ± 2.61	0.5666
CD3+ FOXP3+ T cells, % (as % of all nucleated cells)	0.48 ± 0.09	0.37 ± 0.06	0.3468
Proportion of CD3+ cells co-expressing CD8, %	65.72 ± 3.83	66.11 ± 2.92	0.9362
Proportion of CD3+ cells co-expressing FOXP3, %	29.52 ± 3.57	19.01 ± 3.23	0.0361*
T cells to LMO7+ cells ratio			
CD3+ T cells/LMO7+ cells ratio	0.29 ± 0.08	9.74 ± 6.90	0.1760
CD3+ CD8+ T cells/LMO7+ cells ratio	0.21 ± 0.07	7.37 ± 5.36	0.1865
CD163+ cells, % (as % of all nucleated cells)	7.31 ± 1.13	9.32 ± 1.07	0.2081
GranzymeB expression			
GranzymeB+ cells, %	5.50 ± 0.83	4.66 ± 0.99	0.5218
GranzymeB+ CD3+ CD8+ T cells, %	0.29 ± 0.10	0.64 ± 0.24	0.1732
Proportion of CD3+ CD8+ cells co-expressing GranzymeB, %	19.29 ± 3.64	33.13 ± 4.81	0.0268*
GranzymeB- CD3+ CD8+ T cells, %	1.52 ± 0.47	1.60 ± 0.31	0.8913
Proportion of CD3+ CD8+ cells not co-expressing GranzymeB, %	80.19 ± 3.65	66.87 ± 4.81	0.0331*
Proportion of CD3+ FOXP3+ T cells within a specified distance from LMO7+ cells, %			
Within 25 μm	14.09 ± 3.39	3.12 ± 1.28	0.0040*
Within 50 μm	57.88 ± 6.37	9.35 ± 3.31	0.0000*
Within 100 μm	96.11 ± 8.45	44.30 ± 5.21	0.0000*
Average distance to LMO7+ cells for each CD3+ FOXP3+ T cell, μm	56.21 ± 7.25	125.36 ± 11.76	0.0000*
Average distance to LMO7+ for each CD3+ CD8+ GranzymeB+ T cells, μm	116.59 ± 18.00	51.82 ± 10.35	0.0030*
Average distance to LMO7+ for each CD3+ CD8+ GranzymeB- T cells, μm	60.14 ± 7.18	134.05 ± 16.73	0.0002*
Proportion of unique LMO7 cells, %	9.28 ± 4.20	25.15 ± 4.18	0.0107*

Patients with high LMO7 expression showed a significant increase in the proportion of CD3^+^ cells co-expressing FOXP3 (29.52 ± 3.57%) compared to those with low LMO7 expression (19.01 ± 3.23%; *p* = 0.0361; Fig. 2d). The proportion of CD3^+^ CD8^+^ cells co-expressing GranzymeB was low in the high LMO7-expressing group (19.29 ± 3.64% vs. 33.13 ± 4.81%; *p* = 0.0268; Fig. 2e), and the proportion of CD3^+^ CD8^+^ cells not co-expressing GranzymeB was high in the high LMO7-expressing group (80.19 ± 3.65% vs. 66.87 ± 4.81%; *p* = 0.0331; Fig. 2f). Notably, no significant differences were observed in the total number of nucleated cells, and the percentage of all nucleated cells in CD3^+^ T cells (Fig. 2g), CD3^+^ CD8^+^ T cells (Table 1), CD163^+^ macrophages (Table 1), GranzymeB^+^ cells (Table 1), and CD3^+^ FOXP3^+^ T cells (Table 1) between the groups. Approximately two-thirds of all CD3^+^ lymphocytes co-expressed CD8 in both groups (Fig. 2h). Thus, there was evidence that immune cell infiltration in the PDAC microenvironment was complex, and attention should be focused on both common immune cell populations and changes in subpopulations based on single-cell protein levels.

The proximity and nearest neighbor analysis were further employed to evaluate the spatial relationship between immune and LMO7^+^ cells (Fig. 2i). The average distance from each CD3^+^ FOXP3^+^ cell to an LMO7^+^ cell was closer in patients with high LMO7 expression (56.21 ± 7.25 μm) than in those with low LMO7 expression (125.36 ± 11.76 μm; *p* = 0.0000; Fig. 2j). In patients with high LMO7 expression, the percentage of CD3^+^ FOXP3^+^ T cells located within a 25 μm radius from an LMO7^+^ cell was higher (14.09 ± 3.39%) than in those with low LMO7 expression (3.12 ± 1.28%; *p* = 0.0040). This difference remained statistically significant at both the 50 μm (57.88 ± 6.37% vs. 9.35 ± 3.31%; *p* = 0.0000) and 100 μm (96.11 ± 8.45% vs. 44.30 ± 5.21%; *p* = 0.0000) distances in the high LMO7-expressing group (Table 1).

The average distance to an LMO7^+^ cell for each CD3^+^ CD8^+^ GranzymeB^+^ T cell was greater in the high LMO7-expressing group than in the low LMO7-expression group (116.59 ± 18.00 μm vs. 51.82 ± 10.35 μm; *p* = 0.0030; Fig. 2k). However, the average distance to an LMO7^+^ cell for each CD3^+^ CD8^+^ GranzymeB^-^ T cell was closer in the high LMO7-expressing group than in the other group (60.14 ± 7.18 μm vs. 134.05 ± 16.73 μm; *p* = 0.0002; Fig. 2l). Furthermore, patients with high LMO7 expression had low unique LMO7^+^ cells, that is, LMO7^+^ cells with only a single CD3^+^ FOXP3^+^ cell within a 100 μm radius. This indicates that CD3^+^ FOXP3^+^ cells enriched by LMO7 exhibited clustering rather than dispersion (Fig. 2m).

These results suggest that PDAC tissues with high LMO7 expression exhibited a high proportion of infiltrating CD3^+^ FOXP3^+^ T cells and an increased presence of dysfunctional cytotoxic T cells. CD3^+^ FOXP3^+^ T cells and dysfunctional cytotoxic T cells were closer in spatial proximity to LMO7^+^ cells. Conversely, cytotoxic T cells were farther away from LMO7 cells. Therefore, LMO7 in tumor cells plays a crucial role in regulating Treg and cytotoxic T cells.

### LMO7 facilitated immune evasion by promoting the enrichment of Tregs in PDAC cells and immune-competent mice

We constructed LMO7 knockdown and overexpressing cell lines. In CFPAC-1 cells, all three siRNAs, particularly SiLMO7#1, downregulated LMO7 transcription and protein expression (Fig. S2a, c). SiLMO7#1 was selected for further experiments and referred to as SiLMO7. In Mia Paca-2 cells, the LMO7 overexpression plasmid significantly increased LMO7 transcription and protein levels. (Fig. S2b, d). We isolated naive T cells from human peripheral blood using magnetic bead selection to explore the potentials of human LMO7 in promoting the differentiation of these cells in vitro. The characteristic markers obtained through flow cytometry analysis confirmed the high purity of CD4^+^ CD45RA^+^ Naive T cells (Fig. S2e).

**Fig. 3:**
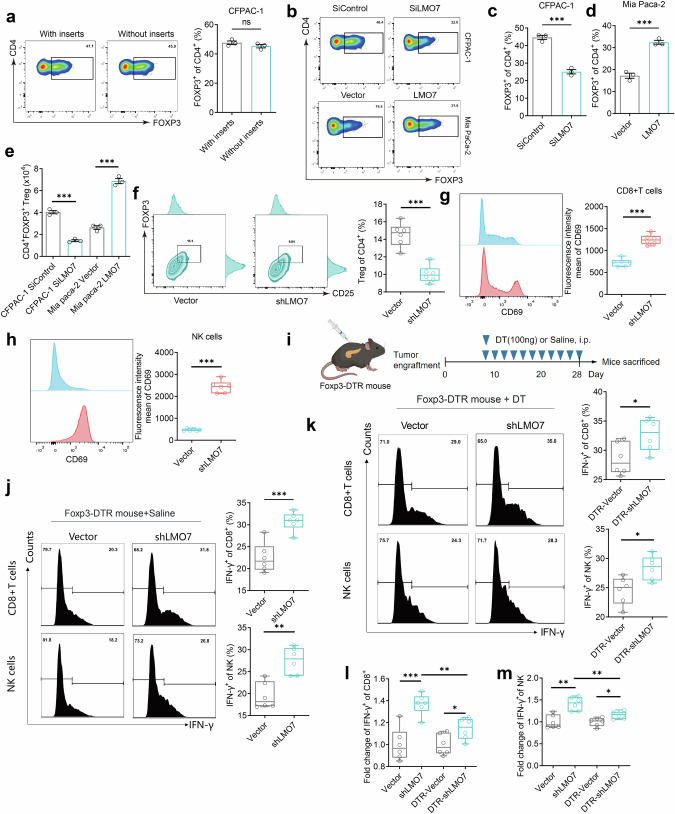
LMO7 facilitates immune evasion by promoting the enrichment of Tregs in PDAC cells and immune-competent mice. **a** Flow cytometry plots (CD45^+^ gated, CD4^+^ FOXP3^+^ of CD4^+^) (left) and the percentages of Tregs (right) after co-culture. Data are presented as mean ± SEM (*n* = 3 independent biological replicates). Flow cytometry plots (**b**) (CD45^+^ gated, CD4^+^ FOXP3^+^ of CD4^+^) and the percentages of Treg differentiation after LMO7 knockdown in CFPAC-1 cells (**c**) and overexpression in Mia Paca-2 cells (**d**). Data are presented as mean ± SEM (*n* = 3 independent biological replicates). **e** Number of Treg chemotaxis after LMO7 knockdown in CFPAC-1 cells and overexpression in Mia Paca-2 cells. Data presented as mean ± SEM (*n* = 3 independent biological replicates). **f** Representative flow cytometry plots (CD45^+^ CD3^+^ CD4^+^ gated, CD4^+^ CD25^+^ FOXP3^+^ of CD4^+^) (left) and the percentages of infiltrating Tregs (right) by constructing an immune-competent mouse orthotopic PDAC model. Data are presented as mean ± SEM. *n* = 6 mice per group. **g** Flow cytometry plots showing infiltrating CD8^+^ T cell CD69 intensity detection (CD45^+^ CD3^+^ CD8^+^ gated). Data are presented as mean ± SEM. *n* = 6 mice per group. **h** Flow cytometry plots demonstrating infiltrating NK cell CD69 intensity detection (CD45^+^ CD3^-^ NK1.1^+^ gated). Data are presented as mean ± SEM. *n* = 6 mice per group. **i** Schematic illustration of inducing the Foxp3-DTR model mice. **j** Flow cytometric analysis of CD8^+^ IFN-γ^+^ T cells (CD45^+^ CD3^+^ gated) and CD3^-^ NK1.1^+^ IFN-γ^+^ NK infiltrating PDAC in Foxp3-DTR mice treated with saline. Data are presented as mean ± SEM. *n* = 6 mice per group. **k** Flow cytometric analysis of CD8^+^ IFN-γ^+^ T cells (CD45^+^ CD3^+^ gated) and CD3^-^ NK1.1^+^ IFN-γ^+^ NK infiltrating PDAC in Foxp3-DTR mice treated with DT. Data are presented as mean ± SEM. *n* = 6 mice per group. **l** Statistical analysis of the relative fold change of CD8^+^ IFN-γ^+^ T cells (CD45^+^ CD3^+^ gated). Data are presented as mean ± SEM. *n* = 6 mice per group. **m** Statistical analysis of the relative fold change of CD3^-^ NK1.1^+^ IFN-γ^+^ NK cells. Data are presented as mean ± SEM. *n* = 6 mice per group. Statistical significance was determined using two-tailed unpaired *t*-test. *p < 0.05; **p < 0.01; ***p < 0.001.

To investigate how PDAC cell-derived LMO7 affected naive T cells, we employed both direct and indirect co-culture models (Fig. S2f). After co-culturing CFPAC-1 and naive T cells for 5 d, collecting suspended cells each day for flow cytometry analysis, differences were observed on the third day, which increased by the fifth day (Fig. S2g). After 5 d of co-culture in both models, there was a differentiation of naive T cells into CD4^+^ FOXP3^+^ Tregs. However, the ratio in both models was not significant (Fig. 3a). This suggested that human PDAC cells with high LMO7 expression can induce the differentiation of naive T cells into Treg cells through an indirect mechanism, and this process may involve the participation of other factors.

Subsequently, we conducted differentiation co-culture experiments with established PDAC cell lines (Fig. 3b). In the CFPAC-1 co-culture model, LMO7 interference significantly inhibited the differentiation of naive T cells into CD4^+^ FOXP3^+^ Treg cells compared to the control group (Fig. 3c). In the Mia Paca-2 co-culture model, overexpression of LMO7 significantly promoted the differentiation of naive T cells into CD4^+^ FOXP3^+^ Treg cells (Fig. 3d). Furthermore, we conducted in vitro studies using magnetic bead-isolated human peripheral blood Treg cells to investigate whether human LMO7 can promote the chemotaxis of Treg cells. The isolated cells were confirmed to be highly pure CD4^+^ FOXP3^+^ Treg cells (Fig. S2h). LMO7 interference inhibited the chemotaxis of Treg cells in the CFPAC-1 chemotaxis model, and LMO7 overexpression significantly promoted the chemotaxis of Treg cells in the Mia Paca-2 chemotaxis model (Fig. 3e). To investigate the impact of LMO7 on the proliferation of Treg cells, we removed the co-culture chambers and extended the co-culture time to 72 h. The results showed that changes in LMO7 did not affect the expression levels of Edu^+^ Treg cells in both CFPAC-1 and Mia Paca-2 co-culture models (Fig. S2i, j).

We further elucidated the in vivo role of LMO7 by constructing an immune-competent mouse orthotopic model of PDAC (Fig. S2k). Tumors were obtained from the mice, dissociated into single cells, and subjected to flow cytometry analysis (Fig. S2l). The results indicated that the LMO7 knockdown significantly reduced the enrichment of CD4^+^ CD25^+^ FOXP3^+^ Treg cells (Fig. 3f) and increased the CD69 expression intensity in infiltrating CD3^+^ CD8^+^ T cells (Fig. 3g) and CD3^-^ NK1.1^+^ NK cells (Fig. 3h).

We established a Foxp3-DTR mouse orthotopic PDAC model (Fig. 3i) and analyzed the IFN-γ^+^ subpopulations of infiltrating CD8^+^ T and NK cells in the Saline group. After LMO7 knockdown, the levels of CD3^+^ CD8^+^ IFN-γ^+^ T and CD3^-^ NK1.1^+^ IFN-γ^+^ NK cells significantly increased (Fig. 3j). This indicates that LMO7 promoted immune evasion in PDAC in vivo. We hypothesized that this immune evasion involved the crucial role of Treg cells. We depleted Foxp3^+^ Treg cells by intermittently injecting diphtheria toxin. In the Foxp3-DTR model, LMO7 knockdown modestly reversed the infiltration levels of CD3^+^ CD8^+^ IFN-γ^+^ T and CD3^-^ NK1.1^+^ IFN-γ^+^ NK cells (Fig. 3k). Additionally, there was a significant difference in the relative changes in cell infiltration levels between the shLMO7 and DTR-shLMO7 groups for CD3^+^ CD8^+^ IFN-γ^+^ T cells and CD3^-^ NK1.1^+^ IFN-γ^+^ NK cells (Fig. 3l, m). This suggests that Treg cells play a crucial role in the immune evasion promoted by LMO7.

### Tumor cells employed LMO7 to promote the differentiation and chemotaxis of Treg cells through TGF-β and facilitated the chemotaxis of Treg cells through CCL5

**Fig. 4:**
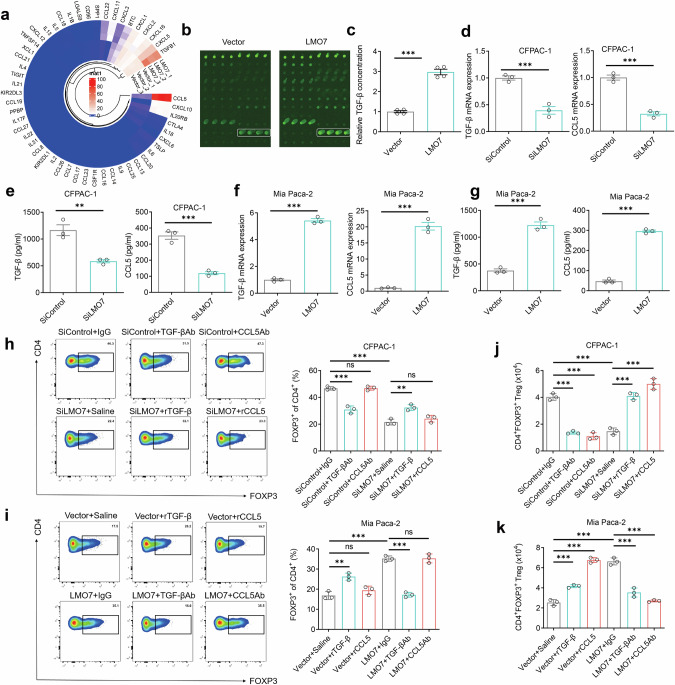
LMO7 promotes the differentiation and chemotaxis of Tregs through TGF-β and facilitates the chemotaxis of Tregs through CCL5. **a** Differential gene convolution heat map. **b** Scanning image of Human Th1/Th2/Th17 Array in Vector and LMO7 Groups. **c** Relative concentration changes of TGF-β protein in cells and their controls. **d** Transcription levels of TGF-β/CCL5 after LMO7 knockdown. **e** Extracellular protein levels of TGF-β/CCL5 after LMO7 knockdown. **f** Transcription levels of TGF-β/CCL5 after LMO7 overexpression. **g** Extracellular protein levels of TGF-β/CCL5 after LMO7 overexpression. **h** Flow cytometric analysis of CD4^+^ FOXP3^+^ Treg in the differentiation models of CFPAC-1 treated with SiControl + IgG, SiControl + TGF-βAb, SiControl + CCL5Ab, SiLMO7 + Saline, SiLMO7 + rTGF-β, and SiLMO7 + rCCL5. **i** Flow cytometric analysis of CD4^+^ FOXP3^+^ Treg in the differentiation model of Mia Paca-2 treated with Vector + Saline, Vector + rTGF-β, Vector + rCCL5, LMO7 + IgG, LMO7 + rTGF-βAb, and LMO7 + CCL5Ab. **j** Quantitative analysis of the chemotaxis model of CFPAC-1 treated with SiControl + IgG, SiControl + TGF-βAb, SiControl + CCL5Ab, SiLMO7 + Saline, SiLMO7 + rTGF-β, and SiLMO7 + rCCL5. **k** Quantitative analysis of the chemotaxis model of Mia Paca-2 treated with Vector + Saline, Vector + rTGF-β, Vector + rCCL5, LMO7 + IgG, LMO7 + rTGF-βAb, and LMO7 + CCL5Ab. Data are presented as mean ± SEM (*n* = 3 independent biological replicates). Statistical significance was determined using two-tailed unpaired *t*-test. *p* < 0.05; **p < 0.01; ***p < 0.001.

To investigate the regulatory mechanisms of LMO7, we conducted RNA sequencing on Vector and LMO7-overexpressing Mia Paca-2 cells. The results revealed a significant increase in TGFB1, CCL5, CXCL5, CXCL10, CXCL11, and IL20RB (Fig. 4a). Gene ontology (GO) enrichment analysis of differentially expressed genes showed that LMO7 was involved in immune system processes and responses (Fig. S2m), consistent with our experimental findings. Additionally, there was a significant enrichment of differentially expressed genes in protein binding functions (Fig. S2m).

To explore the levels of secreted proteins in the TME, we conducted Human Th1/Th2/Th17 Array Q1 array analysis on the conditioned media of Vector and LMO7-overexpressing cells (Fig. 4b). The most notable difference was observed in the tumor-associated TGF-β protein, which significantly increased following LMO7 overexpression (Fig. 4c). We further quantified the extracellular and transcriptional levels of CCL5 and TGF-β using ELISA and PCR, respectively. After knocking down LMO7 in CFPAC-1 cells, the transcription levels of TGF-β and CCL5 decreased (Fig. 4d), along with a reduction in the extracellular protein levels (Fig. 4e). Conversely, in Mia Paca-2 cells, both the transcription and extracellular protein levels of TGF-β and CCL5 significantly increased after LMO7 overexpression (Fig. 4f, g).

Moreover, we conducted rescue experiments for both TGF-β and CCL5 regarding Treg cell differentiation and chemotaxis. In the CFPAC-1 co-culture model, we added IgG, TGF-β antibody, and CCL5 antibody to the SiControl group and added saline, recombinant TGF-β, and recombinant CCL5 to the SiLMO7 group. The results revealed that TGF-βAb effectively suppressed the differentiation of Naive T to CD4^+^ Foxp3^+^ Treg cells, and recombinant TGF-β successfully restored the inhibitory effects on Treg cell differentiation induced by LMO7 knockdown (Fig. 4h). However, no statistically significant differences were observed with CCL5Ab and recombinant CCL5 (Fig. 4h). In the Mia Paca-2 co-culture model, we added saline, recombinant TGF-β, and recombinant CCL5 to the Vector group, and IgG, TGF-β antibody, and CCL5 antibody to the LMO7 group. The results demonstrated that recombinant TGF-β induced the differentiation of naive T to CD4^+^ Foxp3^+^ Treg cells (Fig. 4i). The addition of the TGF-β antibody suppressed the Treg cell differentiation induced by LMO7 overexpression (Fig. 4i). However, no statistically significant differences were observed with CCL5Ab and recombinant CCL5 (Fig. 4i). These results highlight the significant role of TGF-β in LMO7-mediated processes, specifically in naive T cell differentiation to CD4^+^ Foxp3^+^ Treg. However, CCL5 did not influence this differentiation process.

Furthermore, we conducted a recovery experiment for Treg cell chemotaxis. In the chemotaxis model of CFPAC-1, we added IgG, TGF-β antibody, and CCL5 antibody to the SiControl group, and saline, recombinant TGF-β, and recombinant CCL5 to the SiLMO7 group. The results showed that both the TGF-β and CCL5 antibodies inhibited Treg cell chemotaxis. Additionally, the addition of recombinant TGF-β and CCL5 restored the chemotaxis inhibition caused by LMO7 knockdown (Fig. 4j). In the Mia Paca-2 chemotaxis model, we added saline, recombinant TGF-β, and recombinant CCL5 to the Vector group, and IgG, TGF-β antibody, and CCL5 antibody to the LMO7 group. The results showed that both recombinant TGF-β and CCL5 induced Treg cell chemotaxis. Furthermore, the addition of TGF-β and CCL5 antibodies inhibited Treg cell chemotaxis induced by LMO7 overexpression (Fig. 4k). These results indicate that both TGF-β and CCL5 play crucial roles in the LMO7-mediated Treg chemotaxis process. Overall, LMO7 in tumor cells promotes the differentiation and chemotaxis of Treg cells through TGF-β and facilitates the chemotaxis of Treg cells through CCL5.

### Foxp1 served as a crucial link in LMO7-mediated regulation of TGF-β/CCL5 through FBS (Foxp1 binding sites) 2 and FBS I/III

As confirmed by the above experiments, LMO7 was significantly enriched in protein binding functions (Fig. S2m). LMO7 contains multiple domains, with LIM as a common protein-binding domain. We conducted immunoprecipitation-mass spectrometry (IP-MS) on endogenous LMO7 in CFPAC-1 cells to elucidate the downstream regulatory targets of LMO7 due to its function as a binding protein. Additionally, we utilized the UCSC and JASPAR databases for transcriptional predictions of TGF-β and CCL5. The results indicated that Foxp1 and Stat1, two proteins bound to LMO7, were directly involved in the transcriptional regulation of TGF-β/CCL5 (Fig. S2n). According to the mass spectrometry scores, enrichment abundance, and available evidence, we selected Foxp1 as the focus of our study.

**Fig. 5:**
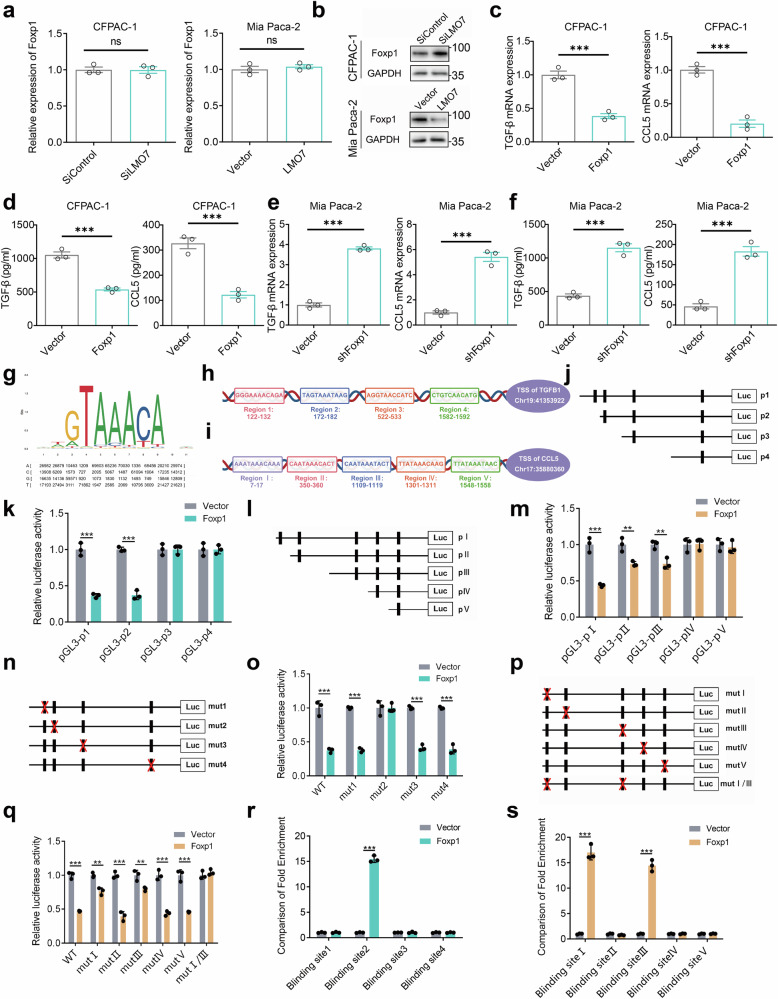
Foxp1 is a crucial link in LMO7-mediated regulation of TGF-β/CCL5. **a** Transcription levels of Foxp1 after LMO7 knockdown (left) and overexpression (right). **b** Protein levels of Foxp1 after LMO7 knockdown (up) and overexpression (down). **c** Transcriptional levels of TGF-β and CCL5 after Foxp1 overexpression. **d** Extracellular protein levels of TGF-β and CCL5 after Foxp1 overexpression. **e** Transcriptional levels of TGF-β and CCL5 after Foxp1 knockdown. **f** Extracellular protein levels of TGF-β and CCL5 after Foxp1 knockdown. **g** Sequence logo and frequency matrix of the transcription factor Foxp1. **h** Regions in the TGF-β promoter where Foxp1 may bind. **i** Regions in the CCL5 promoter where Foxp1 may bind. **j, k** Luciferase activities of TGF-β promoter reporter vectors. Data presented as mean ± SEM (*n* = 3 independent biological replicates). **l**, **m** Luciferase activities of CCL5 promoter reporter vectors. **n**, **o** Luciferase assay for the TGF-β promoter mutation site (Red characters in the binding regions suggest the putative or mutated Foxp1 binding sequences). **p**, **q** Luciferase assay for the CCL5 promoter mutation site (Red characters in the binding regions suggest the putative or mutated Foxp1 binding sequences). ChIP-qPCR assay detecting the binding of Foxp1 to the TGF-β (**r**) and CCL5 (**s**) promoter. Data are presented as mean ± SEM (*n* = 3 independent biological replicates). Statistical significance was determined using two-tailed unpaired *t*-test. *p* < 0.05; **p < 0.01; ***p < 0.001.

LMO7 knockdown and overexpression did not affect Foxp1 transcription levels (Fig. 5a). However, LMO7 knockdown promoted the expression of Foxp1 protein, while its overexpression inhibited Foxp1 protein expression (Fig. 5b). To better understand the role of Foxp1 in TGF-β/CCL5 regulation, we generated stable cell lines with Foxp1 overexpression and knockdown, alongside their corresponding controls. Foxp1 overexpression and knockdown significantly reduced (Fig. 5c, d) and increased (Fig. 5e, f) the mRNA and secreted TGF-β/CCL5 protein levels, respectively.

We identified potential Foxp1 binding sites (FBS) in the promoter region of TGF-β/CCL5 using the JASPAR database (Fig. 5g). Four putative FBS were identified in the TGF-β genomic region (Region 1: 122–132, Region 2: 172–182, Region 3: 522–533, Region 4: 1582–1592) (Fig. 5h), and five putative FBS were identified in the CCL5 genomic region (Region I: 7–17, Region II: 350–360, Region III: 1109–1119, Region IV: 1301–1311, Region V: 1548–1558; Fig. 5i). Luciferase reporter assay with binding site deletion experiments indicated that in CFPAC-1 cells overexpressing Foxp1, FBS 2 of the TGF-β promoter induced Foxp1 to inhibit its promoter activity. This effect was specific to FBS 2 and was not observed in FBS 1, 3, or 4 (Fig. 5j, k). For the CCL5 promoter region, Foxp1 inhibition of promoter activity was induced in FBS I and III, while no such effect was observed in FBS II, IV, or V (Fig. 5l, m). The results of the luciferase reporter assay with binding site mutation experiments also indicated that FBS 2 of the TGF-β promoter and FBS I and III of the CCL5 promoter induced the inhibition of promoter activity by Foxp1 in LMO7-overexpressing PDAC cells (Fig. 5n–q). The ChIP results further indicated that Foxp1 was selectively recruited to the promoter region containing FBS 2 in TGFβ and the promoter regions containing FBS I and FBS III in CCL5 (Fig. 5r, s). Therefore, FBS 2 is crucial for Foxp1-mediated inhibition of TGF-β transcription, and FBS I and FBS III are critical for Foxp1-mediated inhibition of CCL5 transcription.

**Fig. 6:**
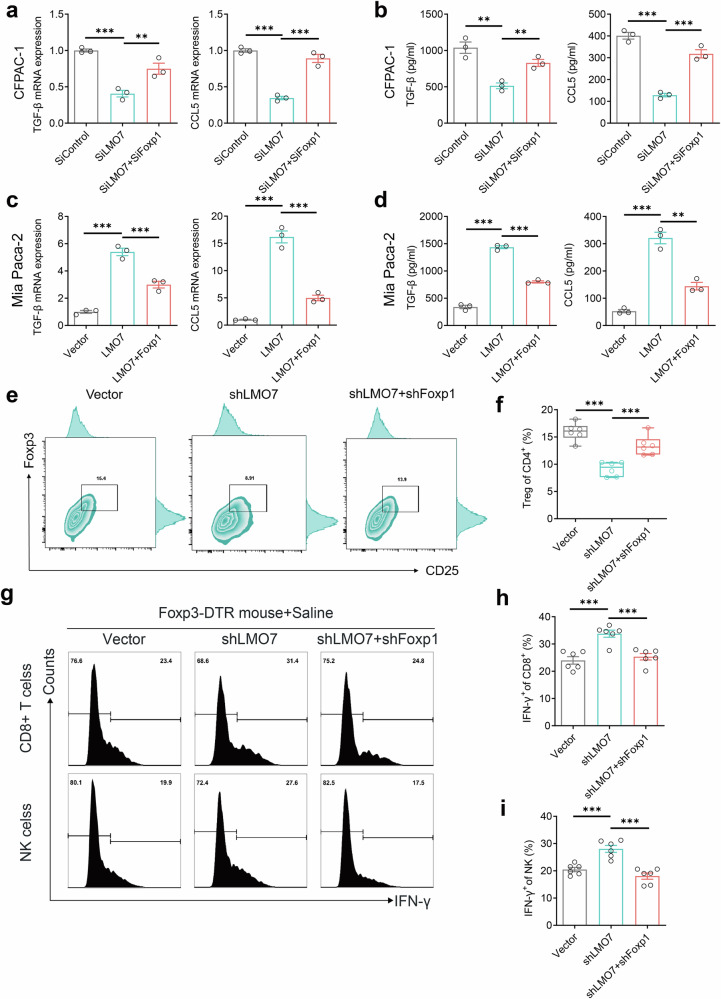
LMO7 promotes immune evasion through Foxp1. **a** Transcription levels of TGF-β and CCL5 after Foxp1 knockdown on the basis of LMO7 interference. Data are presented as mean ± SEM (*n* = 3 independent biological replicates). **b** Extracellular protein levels of TGF-β and CCL5 after Foxp1 knockdown on the basis of LMO7 interference. Data are presented as mean ± SEM (*n* = 3 independent biological replicates). **c** Transcription levels of TGF-β and CCL5 after Foxp1 overexpression based on LMO7 overexpression. **d** Extracellular protein levels of TGF-β and CCL5 after Foxp1 overexpression based on LMO7 overexpression. Data are presented as mean ± SEM (*n* = 3 independent biological replicates). **e** Flow cytometric analysis of infiltrating CD4^+^ CD25^+^ Foxp3^+^ (CD45^+^ CD3^+^ CD4^+^ gated, CD4^+^ CD25^+^ Foxp3^+^ of CD4^+^) Tregs after LMO7 and Foxp1 knockdown. Data are presented as mean ± SEM. *n* = 6 mice per group. **f** Analysis of infiltrating CD4^+^CD25^+^Foxp3^+^ Tregs as a percentage of CD4^+^ T cells. Data are presented as mean ± SEM. *n* = 6 mice per group. **g** Flow cytometric analysis of tumor-infiltrating CD8^+^ T cells and NK cell subpopulations in the Foxp3-DTR model. Data are presented as mean ± SEM. *n* = 6 mice per group. **h** Analysis of infiltrating CD8^+^ IFN-γ^+^ T cells. Data are presented as mean ± SEM. *n* = 6 mice per group. **i** Analysis of infiltrating CD3^-^NK1.1^+^ IFN-γ^+^ NK cells. Data are presented as mean ± SEM. *n* = 6 mice per group. Statistical significance was determined using two-tailed unpaired *t*-test. *p* < 0.05; **p < 0.01; ***p < 0.001.

To investigate whether LMO7’s regulation of TGF-β/CCL5 is dependent on Foxp1, we conducted in vitro rescue experiments. Foxp1 knockdown reversed the inhibitory effects of LMO7 knockdown on TGF-β/CCL5 transcription (Fig. 6a) and extracellular protein levels (Fig. 6b). Conversely, Foxp1 overexpression reversed the promoting effects of LMO7 overexpression on TGF-β/CCL5 transcription (Fig. 6c) and extracellular protein levels (Fig. 6d). We conducted rescue experiments in a C57BL/6 mouse PDAC model, where Foxp1 knockdown reversed the inhibitory effects of LMO7 knockdown on the enrichment of Treg cells (Fig. 6e, f). Additionally, we performed rescue experiments in the Foxp3-DTR mouse + saline model (Fig. 6g). The results indicated that Foxp1 knockdown reversed LMO7’s inhibitory effects on the infiltration levels of CD8^+^ IFN-γ^+^ T cells (Fig. 6h) and CD3-NK1.1^+^ IFN-γ^+^ NK cells (Fig. 6i), suppressing immune evasion.

### LMO7 directly bound to Foxp1 through the LIM domain, regulating immune evasion and promoting its ubiquitination degradation

**Fig. 7:**
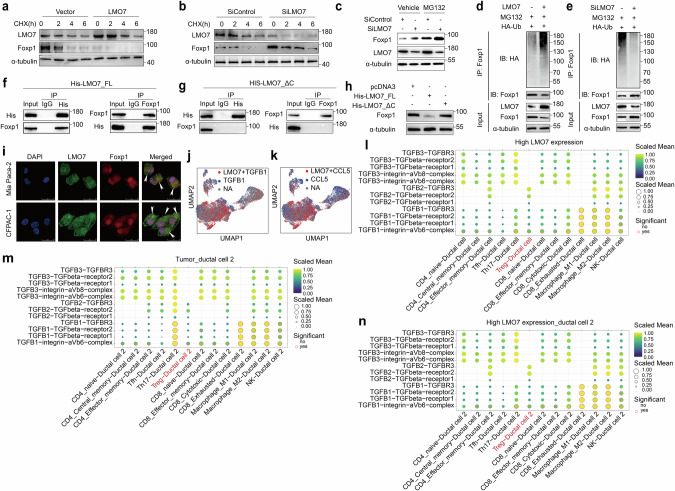
LMO7 promotes ubiquitination degradation of Foxp1. **a** Protein expression of Foxp1 in Mia Paca-2 cells with LMO7 overexpression and control group after cycloheximide treatment. **b** Protein expression of Foxp1 in CFPAC-1 cells with LMO7 knockdown and control group after cycloheximide treatment. **c** Protein level changes of Foxp1 after MG132 treatment in SiControl and SiLMO7 group. **d** Foxp1 was knocked down, and ubiquitin-conjugated Foxp1 was identified via immunoblotting using an anti-HA antibody in LMO7-overexpressed Mia Paca-2 cells and control. **e** Foxp1 was knocked down, and ubiquitin-conjugated Foxp1 was identified through immunoblotting using an anti-HA antibody in CFPAC-1 cells with LMO7 knockdown and control. Mia Paca-2 cells were transfected with a HIS-LMO7_FL (**f**) and HIS-LMO7_ΔC (**g**) plasmid. After 24 h, cell lysates were collected for immunoprecipitation and subsequent western blotting analysis. **h** Foxp1 protein expression was determined in HIS-LMO7_FL or HIS-LMO7_ΔC Mia Paca-2 cells. **i** Confocal microscopy showing colocalization of LMO7 (green) with Foxp1 (red). Scale bars, 25 µm. **j** UMAP plots displaying co-expression analysis of LMO7 and TGFB1. **k** UMAP plots displaying co-expression analysis of LMO7 and CLL5. **l** Dot plot of receptor–ligand interactions among ductal cells and immune cells in PDAC with high LMO7 expression using CellphoneDB. **m** Dot plot of receptor–ligand interactions among ductal cell 2 and immune cells in PDAC using CellphoneDB. **n** Dot plot of receptor–ligand interactions among ductal cell 2 and immune cells in PDAC with high LMO7 expression using CellphoneDB. *n* = 3 independent biological replicates.

The results above indicated that LMO7 in PDAC cells could reduce Foxp1 protein levels (Fig. 5b) and not regulate Foxp1 transcription levels (Fig. 5a). This suggests that LMO7 may influence the stability of Foxp1 protein. LMO7 contains multiple protein-protein interaction domains, including an F-box motif. This motif can recruit substrates to the Skp-Cullin-F-box E3 ligase complex for degradation. When treated with the protein synthesis inhibitor cycloheximide, LMO7 overexpression significantly promoted the degradation of Foxp1 (Fig. 7a). Conversely, Foxp1 degradation was markedly inhibited in LMO7 knockdown cells (Fig. 7b). Additionally, treatment with the proteasome inhibitor MG132 in both SiControl and SiLMO7 PDAC cells restored Foxp1 levels (Fig. 7c). Thus, LMO7 could regulate the stability of Foxp1 protein through a proteasome-dependent pathway.

The ubiquitin-proteasome system (UPS) promotes the proteasomal degradation of its target proteins. Therefore, we investigated whether LMO7 regulates the degradation of Foxp1 protein through UPS. We co-transfected PDAC cells with either LMO7 overexpression or LMO7 knockdown along with a plasmid encoding HA-tagged ubiquitin. The results showed that LMO7 overexpression in Mia Paca-2 cells exhibited a significant increase in polyubiquitination levels (Fig. 7d). Conversely, LMO7 knockdown in CFPAC-1 cells showed a significant decrease in polyubiquitination levels (Fig. 7e). These findings suggest that LMO7 promotes polyubiquitination and proteasomal degradation of Foxp1.

The LIM domain of LMO7 is a well-known protein interaction domain. The C-terminal LIM domain of LMO7 can interact with cellular functional proteins, such as β-catenin. We mutated the C-terminus of LMO7’s LIM domain (Lmo7ΔC). The results showed that in PDAC cells transfected with the full-length LMO7 plasmid (HIS-LMO7_FL), LMO7 could immunoprecipitate with Foxp1 (Fig. 7f). Conversely, in PDAC cells transfected with the HIS-LMO7_ΔC plasmid, the protein marked with HIS antibody could not immunoprecipitate with Foxp1 protein (Fig. 7g). In PDAC cells, overexpression of full-length LMO7 significantly inhibited the expression of Foxp1, whereas LMO7ΔC failed to achieve this effect (Fig. 7h).

Additionally, confocal microscopic analysis of CFPAC-1 and Mia Paca-2 cells revealed colocalization of LMO7 and Foxp1 (Fig. 7i). Due to the crucial roles of LMO7, TGF-β, and CCL5 in PDAC immune evasion, we further analyzed the expression at the single-cell level in PDAC tissues. The results revealed a significant co-expression of LMO7 and TGFB1 in PDAC tissues (Fig. 7j). Notably, in individual cells with high expression of LMO7, TGFB1 expression was prominently observed. Similar observations were made for LMO7 and CCL5 co-expression (Fig. 7k). These data collectively suggest that the C-terminal LIM domain of LMO7 is necessary for the interaction and degradation of Foxp1.

CellChat and CellPhone were employed to dissect the complex ligand-receptor interactions within the TME. Our analysis identified TGF-β signaling as a critical player in the interactions between ductal cells and Tregs in tumors with high LMO7 expression (Fig. S3a). We observed strong TGF-β1 ligand-receptor interactions between ductal cells and Tregs in tissues with high LMO7 expression (Fig. 7l), suggesting a potential paracrine signaling effect from the ductal cells to the Tregs. Notably, this interaction was absent in both normal tissues or in tissues with low LMO7 expression (Fig. S3b, c). Further stratification of ductal cells revealed distinct subtypes (Fig. S3d), with LMO7 predominantly expressed in Ductal cell 2 (Fig. S3e), which is primarily derived from tumor tissues (Fig. S3f, g). We found that Ductal cell 2 demonstrated significant TGF-β signaling interactions with Tregs in both the entire cohort of tumors and specifically within the subset of tumors characterized by high LMO7 expression (Fig. 7m, n). In contrast, such interactions were not significant in tumors with low LMO7 expression (Fig. S3h) and absent in normal tissues. Interestingly, the TGF-β signaling pathway between Ductal cell 1 and Tregs was not significant in either tumor samples (regardless of LMO7 expression levels) or normal samples (Fig. S3i-l).

### LMO7 inhibition combined with TGF-β/CCL5 antibody treatment exerted synergistic effects in reversing PDAC immune evasion and extending survival

**Fig. 8:**
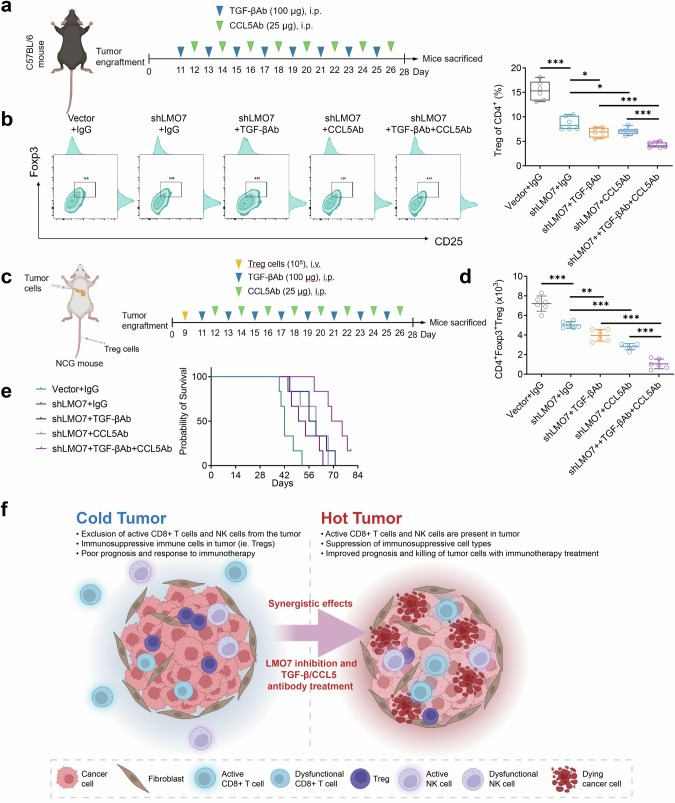
Synergistic effects of LMO7 knockdown and TGF-β/CCL5 antibody treatment in mice. **a** Schematic representation of the treatment strategy for orthotopic tumors. C57BL/6 mice were implanted with Panc02 cells as orthotopic tumors and were treated with TGF-β mAb, CCL5 mAB, and IgG isotype control. **b** Flow cytometric analysis of infiltrating CD4^+^ CD25^+^ Foxp3^+^ Tregs (CD45^+^ CD3^+^ CD4^+^ gated) in mouse PDAC. Data presented as mean ± SEM. *n* = 6 mice per group. **c** Schematic diagram of the therapeutic strategy for orthotopic tumors in NOG-Prkdc^em26Cd52^il2rg^em26Cd22^ (NCG) mice, followed by intravenous injection of Tregs 9 d later. Subsequent treatments were performed with TGF-β antibody, CCL5 antibody, and IgG. **d** Flow cytometric analysis of infiltrating CD4^+^ Foxp3^+^ Tregs in mouse PDAC. Data are presented as mean ± SEM. *n*  = 6 mice per group. **e** Kaplan–Meier survival curves of C57BL/6 mice with the Panc02 cells orthotopic tumors. *n* = 6 mice per group. **f** Schematic diagram of synergistic therapy reversing the cold tumor microenvironment in PDAC. *P* value was determined using unpaired two-sided Student’s *t*-test or log-rank test. *p < 0.05; **p < 0.01; ***p < 0.001.

Next, we investigated whether the combination of LMO7 knockdown and TGF-β/CCL5 antibody treatments synergistically reversed PDAC immune evasion and extended survival. C57BL/6 mice were treated with TGF-β/CCL5 antibodies or IgG isotype control antibodies after inoculation with LMO7 knockdown or control Panc02 cells (Fig. 8a). The combination of shLMO7 with either TGF-β antibody or CCL5 antibody significantly reversed the enrichment of CD4^+^ CD25^+^ Foxp3^+^ Treg cells compared to shLMO7 alone (*p* < 0.05, *p* < 0.05). When shLMO7 was combined with TGF-β + CCL5 antibodies, it exhibited superior reversal capability in Treg enrichment, significantly outperforming the combination of shLMO7 with either TGF-β or CCL5 antibodies (*p* < 0.001, *p* < 0.001; Fig. 8b).

By establishing the xenograft orthotopic PDAC model in NOG-Prkdc^em26Cd52^il2rg^em26Cd22^/Nju (NCG) mice and co-reconstruction with Treg cells (Fig. 8c), we observed that compared to using shLMO7 alone, the combination of shLMO7 with either TGF-β antibody or CCL5 antibody significantly inhibited the chemotaxis of Treg cells (*p* < 0.01, *p* < 0.001). Moreover, the treatment combining shLMO7 with TGF-β + CCL5 antibodies was significantly more effective than the combination of shLMO7 with either TGF-β or CCL5 antibodies (*p* < 0.001, *p* < 0.001; Fig. 8d).

As shown in Fig. 8e, the combination of shLMO7 with TGF-β or CCL5 antibodies extended the mouse survival compared to the single use of shLMO7, although this was not significant (*p* = 0.2034, *p* = 0.2361). However, when shLMO7 was combined with TGF-β + CCL5 antibodies, there was a significant extension in mouse survival compared to shLMO7 alone (*p* = 0.0031). Moreover, the combination of shLMO7 with TGF-β + CCL5 antibodies was better than shLMO7 combined with TGF-β (*p* = 0.0282) or CCL5 (*p* = 0.0210) antibodies (Table. S1). Overall, LMO7 knockdown combined with TGF-β/CCL5 antibody treatment has synergistic effects in reversing PDAC immune evasion and extending mouse survival (Fig. 8f).

## Discussion

LMO7 exhibits abnormal expression across various tumor types and seems to possess tumor-type-specific properties, as indicated by available studies [37–41]. We have previously reported that the upregulation of LMO7 promotes PDAC malignant progression [42]. In this study, we, for the first time, confirmed that PDAC cells utilized LMO7 to enrich Treg cells and dysfunctional cytotoxic T cells, thereby promoting immune evasion. Specifically, LMO7, through its LIM domain, directly bound and promoted the ubiquitination and degradation of Foxp1. Foxp1 negatively regulated TGF-β and CCL5 expression by binding to the promoter regions of these two genes. In in vivo experiments, we found that LMO7 knockdown combined with TGF-β/CCL5 antibody treatment had synergistic effects in reversing PDAC immune evasion and extending survival. Therefore, the LMO7-Foxp1-TGF-β/CCL5 axis could serve as a potential target for immunotherapy in PDAC.

In this study, through the PDAC TCGA and scRNA-seq dataset, we not only demonstrated a close association between high expression of LMO7 and immune suppression but also found that the expression of LMO7 was predominantly in PDAC ductal cells. Although the precise mechanism underlying the impact of heightened LMO7 expression in PDAC cells remains unclear, targeting LMO7 may be effective in clinical applications, as elevated LMO7 expression is associated with a poor prognosis for patients with PDAC. There is a lack of research on the role of LMO7 in tumor immunity; however, a recent study on inflammatory bowel disease suggested that LMO7 could be detected in macrophages and was involved in macrophage activation [45]. Similarly, a study found that LMO7 participated in the modulation of Treg cells in tears [46]. These results suggest that LMO7 was likely involved in the differentiation and regulation of immune cells. Therefore, the role of LMO7 in immune infiltration in tumors is worth exploring.

Through single-cell spatial proteomics based on mIHC, the significant heterogeneity of the PDAC microenvironment was once again confirmed. Although LMO7 did not promote an increase in the percentages of CD3^+^ T cells, CD3^+^ CD8^+^ T cells, CD163^+^ macrophages, GranzymeB^+^ cells, and CD3^+^ FOXP3^+^ T cells among all nucleated cells, it induced Treg and dysfunctional CD8^+^ T cells to be in closer proximity to tumor cells. Moreover, cytotoxic CD8^+^ T cells were observed to reside at a greater distance from the tumor. Specifically, patients with PDAC and high LMO7 expression had a significantly higher proportion of CD3^+^ FOXP3^+^ T cell subpopulation than those with low expression. Additionally, there was a trend towards a decrease in CD3^+^ CD8^+^ GranzymeB^+^ T cells and an increase in CD3^+^ CD8^+^ GranzymeB^-^ T cells in this group. These findings suggest that in the LMO7 high-expression group, dysfunctional T cells were predominant.

Notably, we utilized spatial proteomics analysis to study the spatial distribution of immune cells around each LMO7^+^ PDAC cell. Compared with the low LMO7 group, the high LMO7-expressing group exhibited higher proportions of infiltrating CD3^+^ FOXP3^+^ T cells, CD163^+^ macrophages, and cytotoxic T cells. Moreover, their spatial distribution was noticeably closer to that of LMO7^+^ cells, suggesting the active migration of immune cells and a reflection of the patient’s immune system function. In the high LMO7-expressing group, the average distance from each CD3^+^ FOXP3^+^ T cell to an LMO7^+^ cell was closer, and the proportion of unique LMO7 cells was lower. This reflects the tendency of CD3^+^ FOXP3^+^ T cells to aggregate in clusters within LMO7^+^ cells. Compared to patients with low LMO7 expression, those with high LMO7 expression exhibited an increased average distance of CD3^+^ CD8^+^ GranzymeB^+^ T cells from LMO7^+^ cells. In contrast, compared to the Low LMO7 group, the average distance of CD3^+^ CD8^+^ GranzymeB^-^ T cells from LMO7^+^ cells in the high LMO7-expressing group was closer, suggesting that dysfunctional cytotoxic T cells migrate toward LMO7^+^ cells.

To elucidate the mechanisms by which PDAC cells utilize LMO7 to mediate the enrichment of Treg cells and dysfunctional cytotoxic T cells, we conducted experiments, including RNA sequencing and the Th1/Th2/Th17 Array. We found that LMO7 significantly influences the expression of various cytokines, with TGF-β and CCL5 showing significant differences. These factors can promote the differentiation of naive T cells into Treg cells or enhance the chemotactic ability of immune cells. We validated the regulatory effect of LMO7 on TGF-β and CCL5 mRNA and protein levels using PCR and ELISA. Interestingly, through analysis of scRNA-seq data, we discovered a positive correlation between LMO7 and TGF-β/CCL5 within PDAC tissue samples, aligning with in vitro experimental results. Therefore, we hypothesized that PDAC cells possess a mechanism that upregulates the expression of TGF-β/CCL5 through LMO7, thereby playing a crucial role in the enrichment of Tregs in the PDAC microenvironment. Consistent with our findings, Moo-Young et al. observed that mouse PDAC cells induced the differentiation of naive T cells to Treg cells through the secretion of TGF-β, both in vitro and in vivo [47]. Similarly, it has been indicated that CCL5 plays a significant chemotactic role in recruiting Treg cells in breast cancer and PDAC [48, 49]. We delved into the intricate network of cell-cell interactions facilitated by ligand-receptor pairs. Our analysis, grounded in scRNA-seq data, also unveiled the TGF-β pathway as a pivotal axis in the crosstalk between tumor cells and Tregs.

Furthermore, through systematic bioinformatics analysis and protein mass spectrometry, we focused on the potential transcription factor Foxp1 that might be involved in the regulation of TGF-β/CCL5 gene promoter regions. Foxp1 has been found to bind to the upstream region of the β-adrenergic receptor (β-AR) promoter, inhibiting its expression, controlling brown/beige adipocyte differentiation, and promoting the progression of bladder cancer [50, 51]. Neyroud et al. discovered that Foxp1 is a novel suppressor of skeletal muscle gene expression. In cancer-associated cachexia, the expression of this gene increases, inhibiting MEF2 transcriptional activity and inducing skeletal muscle wasting and weakness [52]. The study by Wang et al. revealed that FOXP1 and PBRM1 bind to the enhancer region of PD-L1, suppressing its expression. Furthermore, EBV-miR-BART11 and EBV-miR-BART17-3p inhibit FOXP1 and PBRM1, respectively, enhancing PD-L1 transcription and promoting immune escape in tumors [53]. In cervical cancer, NAT10/ac4C/FOXP1 induced the expression of GLUT4 and KHK, thereby promoting glycolytic metabolism and immune suppression [54]. In summary, existing literature has consistently reported the close association of Foxp1’s function with immune regulation. Consistent with literature reports, our experimental results demonstrated the specific binding of Foxp1 to the promoter regions of TGF-β/CCL5, inhibiting their activity. This was further validated through ChIP experiments, confirming the specific binding sites of Foxp1 on the target gene promoter regions. Through the construction of in vitro experimental models and a series of experiments, we demonstrated that LMO7 relieved the direct inhibition of TGF-β/CCL5 by ubiquitinating and degrading Foxp1, particularly in a manner dependent on the C-terminal LIM domain of LMO7, thereby promoting Treg cell differentiation and chemotaxis. Furthermore, we confirmed that PDAC cells utilized LMO7 to decrease Foxp1 protein levels in vivo, promoting the secretion of TGF-β/CCL5 and Treg cell infiltration within the tumor tissue. Hence, we demonstrated, for the first time, that PDAC cells utilize LMO7 to regulate TGF-β/CCL5 by ubiquitinating and degrading Foxp1, playing a crucial role in facilitating the formation of an immunosuppressive microenvironment in PDAC.

Additionally, the in vivo experimental results, utilizing the innovatively constructed mouse immune-reconstituted chemotaxis model, confirmed the crucial role of PDAC cells in utilizing LMO7 for Treg cell chemotaxis. Tregs express various chemokine receptors. Chemokine gradients, including CCR4-CCL17/22, CCR5-CCL5, CCR8-CCL1, and CCR10-CCL28, play a role in recruiting Tregs into TME [55–57]. Tregs have been observed to migrate towards tumors, driven by the chemokine gradient produced by tumors. The inhibition of Tregs migration to TME represents a promising strategy for tumor immunotherapy. In melanoma, CCR4 is essential for guiding Tregs to emerging tumor locations from lymph nodes. The signaling of BRAFV600E in melanocytes regulates the production of chemokines associated with CCR4, influencing the recruitment of Tregs to skin sites induced by tumors. Inhibiting the migration of Tregs enhances immunosurveillance in this context [58]. Mogamulizumab, an anti-CCR4 antibody that has been de-fucosylated, lowers the levels of CCR4^+^ T cells and CCR4^+^ Tregs in individuals with cutaneous T-cell lymphoma [59]. Impeding the CCL3-CCR1/CCR5 and CXCL12-CXCR4 axes hindered the accumulation of Tregs in the leukemia-associated hematopoietic microenvironment and slowed down the progression of leukemia [60]. Colorectal cancer (CRC) cells releasing CCL20 attracted Tregs, fostering chemoresistance through FOXO1/CEBPB/NF-κB signaling, which could be a promising strategy for treating CRC [61]. Additionally, Treg infiltration, dependent on CCR4 and CCR5, has been reported in breast cancer, lymphoma, squamous cell carcinoma, and PDAC [62–65]. This implies that further investigation into the targeting of chemokines to impede Treg recruitment should be conducted, particularly in specific populations of patients with PDAC.

Finally, we explored the impact of targeting LMO7 in combination with TGF-β/CCL5 antibody treatment on immune evasion in PDAC. We found that either LMO7 interference or TGF-β/CCL5 antibody treatment alone could partially inhibit immune escape in PDAC and extend the survival of mice. However, when used in combination, the therapeutic effect was more pronounced, leading to a significant extension of survival in mice. Simultaneously, the combination treatment effectively suppressed the infiltration of Treg cells into tumor tissues and enhanced the ratio of CD8^+^ T cells/NK cells and levels of IFN-γ. This suggests that the combination of LMO7 inhibition with TGF-β/CCL5 antibodies can effectively overcome immune evasion in PDAC and activate the immune response. A recent clinical trial published in *Nature* reveals that the personalized RNA neoantigen vaccine stimulates T cells in PDAC, inducing a high-intensity IFN-γ response and promoting the expansion of polyfunctional neoantigen-specific effector CD8^+^ T cells [66]. This finding strengthens the prospects for the future of combination immunotherapy in PDAC.

While LMO7 is primarily expressed in ductal cells, the analysis of single cell sequencing data showed its significant presence in fibroblast cells as well. This observation suggests that fibroblast cells, through their expression of LMO7, may actively participate in the complex interactions within the tumor microenvironment. The expression of LMO7 in these cells could potentially influence the behavior of neighboring cells and contribute to the desmoplastic reaction, a hallmark of PDAC. We acknowledge the need for further investigation to fully understand the functional role of LMO7 in fibroblast cells and its broader impact on PDAC progression. Future studies should explore the mechanistic links between LMO7 expression and the tumor microenvironment to uncover novel therapeutic targets.

In conclusion, our study unveils the LMO7-Foxp1-TGF-β/CCL5 axis in regulating PDAC immune evasion, providing novel insights for innovative PDAC treatment strategies (Fig. Graphical abstract). We discovered that targeted therapy combining LMO7 inhibition with TGF-β/CCL5 antibodies effectively overcomes immune evasion and prolongs mouse survival. This combined treatment addresses both the tumor itself and immune-suppressive factors in the TME, resulting in a synergistic anti-tumor effect. We propose LMO7 as a novel therapeutic target for PDAC, and disrupting its expression or function can enhance the effectiveness of immunotherapy. Our findings provide novel insights and potential targets for immunotherapy and offer a crucial reference for exploring the mechanisms underlying immune evasion in PDAC. However, extensive research and clinical trials are essential for further validation and optimization of this therapeutic strategy.

## Supplementary information

Supplementary information

Supplementary Materials-Original western blots

aj-checklist

## Data Availability

The data analyzed during this study are included in this published article and the supplemental data files. This study did not generate new raw scRNA-seq data. The raw scRNA-seq data we used to extend our analyses on T cells has been uploaded to the Genome Sequence Archive (https://ngdc.cncb.ac.cn/gsa/) under project PRJCA001063 and accession number GSA: CRA001160. TCGA datasets for PAAD were all obtained from UCSC Xena (https://xenabrowser.net/datapages/). This paper does not report original code. Any additional information required to reanalyze the data reported in this paper is available from the corresponding authors upon reasonable request.
