# Supplementary material for: LIM domain only 7: a novel driver of immune evasion through regulatory T cell differentiation and chemotaxis in pancreatic ductal adenocarcinoma
Source: Cell Death Differ. 2024 Aug 14;32(2):271–90. doi: 10.1038/s41418-024-01358-7 (PMC11803110; doi:10.1038/s41418-024-01358-7)
Supplement: Supplementary file 1 — Supplementary information [file 41418_2024_1358_MOESM1_ESM.pdf]

Supplemental information

Table S1 Combination of shLMO7 with TGF-β/CCL5 antibodies significantly prolongs PDAC mouse survival.

**Groups**	**Vector+IgG VS shLMO7+IgG**	**shLMO7+IgG VS shLMO7+TGF-βAb**	**shLMO7+IgG VS shLMO7+CCL5Ab**	**shLMO7+IgG VS shLMO7+TGF-βAb+CCL5Ab**	**shLMO7+TGF-βAb VS shLMO7+TGF-βAb+CCL5Ab**	**shLMO7+CCL5Ab VS shLMO7++TGF-βAb+CCL5Ab**
**P values**	0.0331*	0.2034	0.2361	0.0031**	0.0282*	0.021*

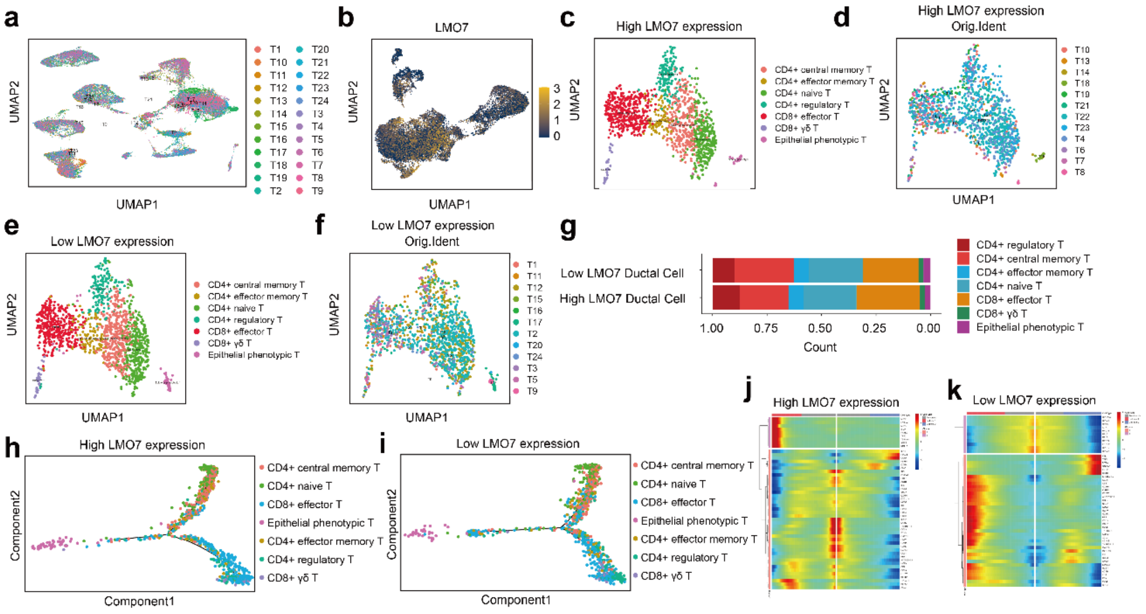

Fig. S1 Relationship between LMO7 and T-cell composition. a UMAP plots showing the transcription heterogeneity of cell clusters colored by sample types in PDAC. **b** UMAP plots showing LMO7 expression levels in ductal cells. **c** Analysis of T-cell composition in tissues with high LMO7 expression. **d** Source distribution of T-cell samples in tissues with high LMO7 expression. **e** Analysis of T-cell composition in tissues with low LMO7 expression. **f** Source distribution of T-cell samples in tissues with low LMO7 expression. **g** Comparison of T-cell composition between tissues with high and low LMO7 expression. **h, i** Pseudo-time of T cell inferred by Monocle2 in high (**h**) and low (**i**) LMO7 expression groups. Each point corresponds to a single cell. Clusters information was shown. **j, k** Analysis of T-cell pseudo-time in tissues with high

(j) and low **(k)** LMO7 expression.

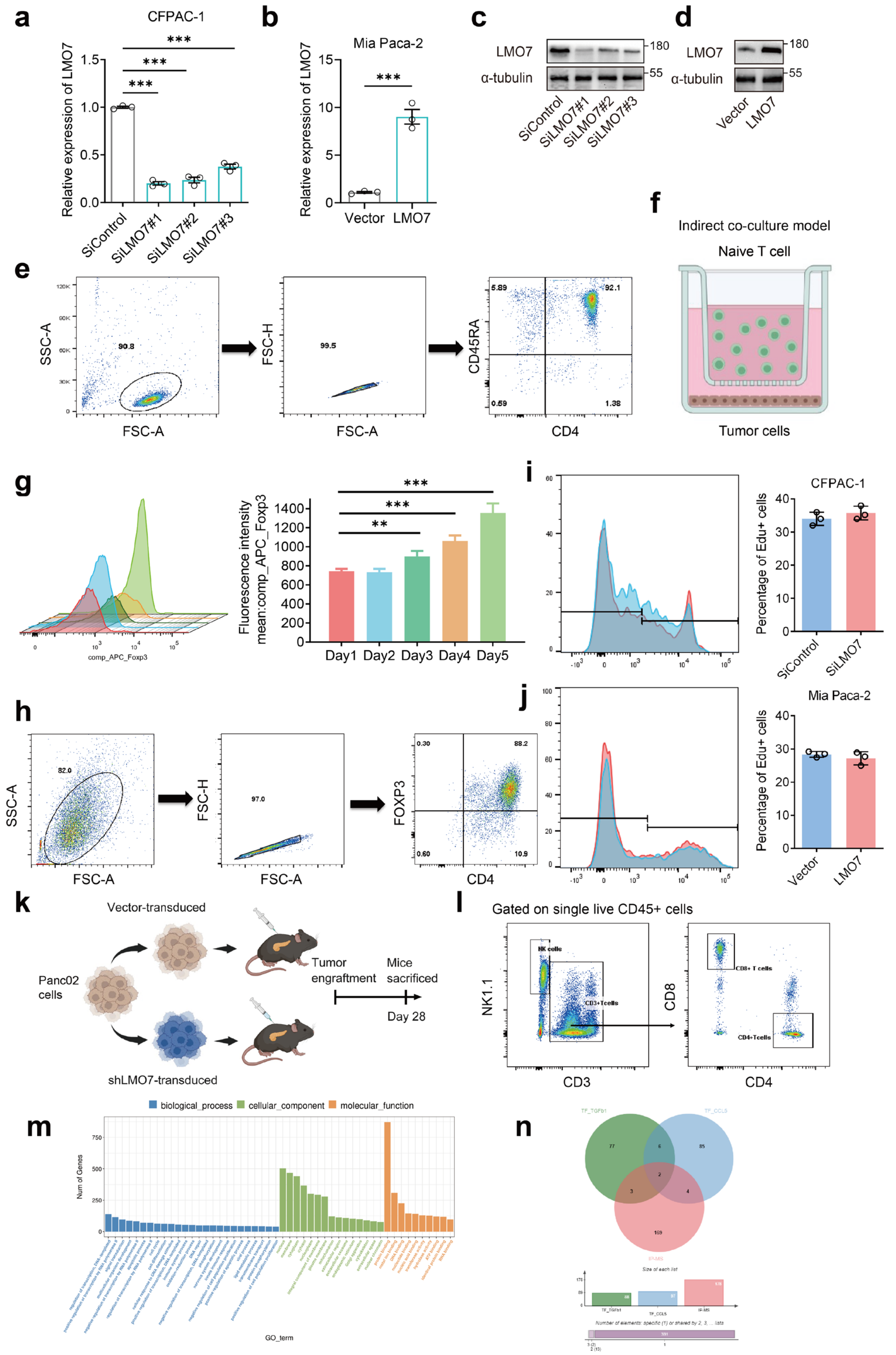

Fig. S2 LMO7 plays a crucial role in the differentiation and chemotaxis of Tregs.

a, c CFPAC-1 cells were infected with SiControl, SiLMO7#1, SiLMO7#2, and

SiLMO7#3, and LMO7 mRNA **(a)** and protein **(c)** levels were validated using qPCR and western blotting with α-tubulin as control. **b, d** qPCR and western blotting confirmed the LMO7 mRNA **(b)** and protein **(d)** levels overexpression efficiency of the LMO7 plasmid in Mia Paca-2 cells. **e** Flow cytometry validation of $CD4^{+}CD45RA^{+}$ cell purity. **f** Diagram illustrating the indirect co-culture method. **g** Time-dependent induction of Treg transformation by CFPAC-1 cells. **h** Flow cytometric verification of the purity of $CD4^{+}FOXP3^{+}$ cells. **i, j** In vitro co-culture systems of CFPAC-1 **(i)** and Mia Paca-2 **(j)** with Tregs for EDU detection. **k** PDAC orthotopic tumors transplantation mouse model. **l** Gating strategy for flow cytometric analysis of infiltrating immune cells. **m** Gene Ontology (GO) enrichment analysis of differentially expressed genes. **n** Venn diagram depicting the overlap in transcription factor predictions for TGF-β, CCL5, and IP-MS. Data are presented as mean ± SEM (n = 3 independent biological replicates).

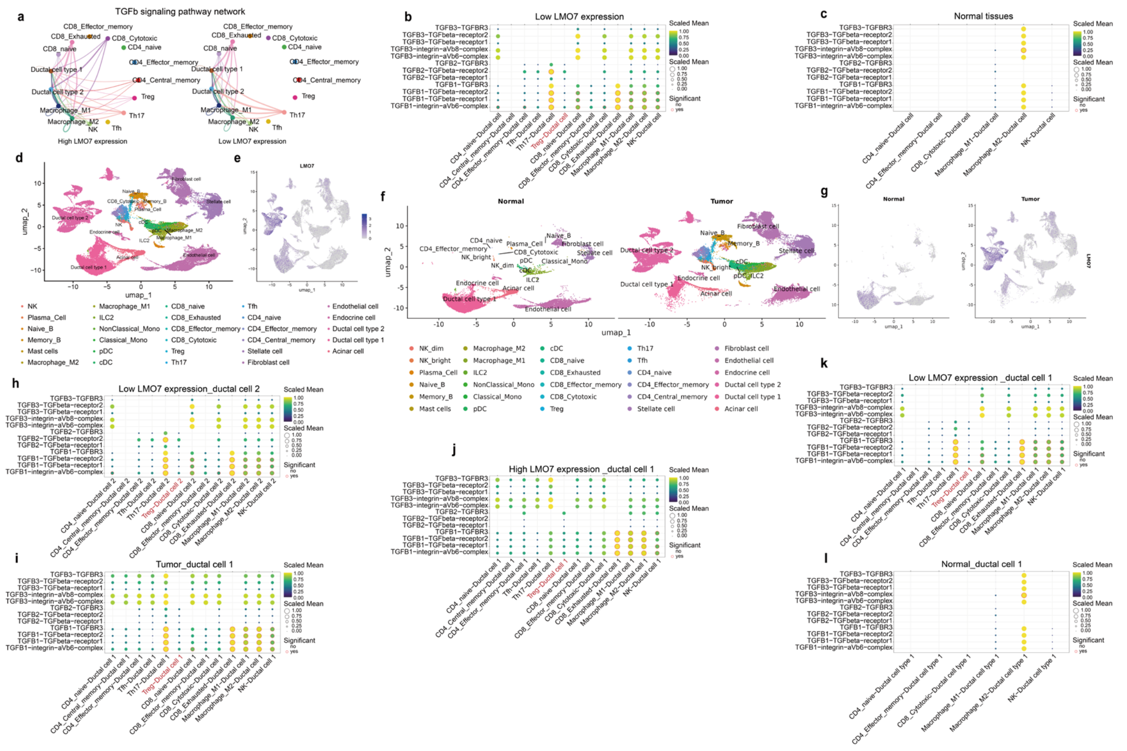

Fig. S3 TGF-β signaling as a critical player in the interactions between ductal cells and Tregs. a Inferred interaction of TGF-β signaling network between ductal cells and immune cells quantified by CellChat. Circle sizes represent the number of cells, and edge width represents communication probability. **b, c** Dot plot of receptor–ligand interactions among ductal cells and immune cells in PDAC with low LMO7 expression **(b)** and normal tissues **(c)** using CellphoneDB. **d** UMAP plots depicting major cell types based on single-cell sequencing data (CRA001160). **e** UMAP plots displaying LMO7 expression of diverse cell types. **f** UMAP plots depicting major cell types in normal and PDAC tissues. **g** UMAP plots displaying LMO7 expression of diverse cell types in normal and PDAC tissues. **h** Dot plot of receptor–ligand interactions among ductal cell 2 and immune cells in PDAC with low LMO7 expression. **i** Dot plot of receptor–ligand interactions among ductal cell 1 and immune cells in PDAC. **j, k** Dot plot of receptor–ligand interactions among ductal cell 1 and immune cells in PDAC with high **(j)** and

low **(k)** LMO7 expression. **l** Dot plot of receptor–ligand interactions among ductal cell 1 and immune cells in normal tissues.
