# Supplementary figures and images for: LIM domain only 7: a novel driver of immune evasion through regulatory T cell differentiation and chemotaxis in pancreatic ductal adenocarcinoma
Source: Cell Death Differ. 2024 Aug 14;32(2):271–90. doi: 10.1038/s41418-024-01358-7 (PMC11803110; doi:10.1038/s41418-024-01358-7)

**Fig. 5**

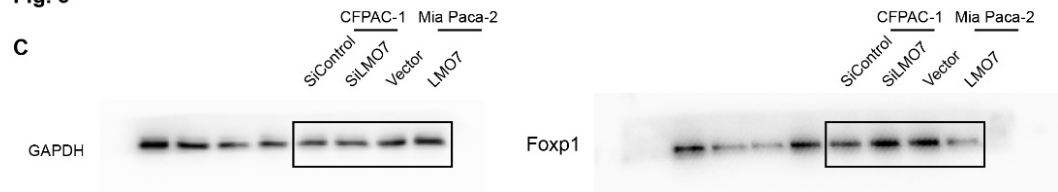

**Fig. 7**

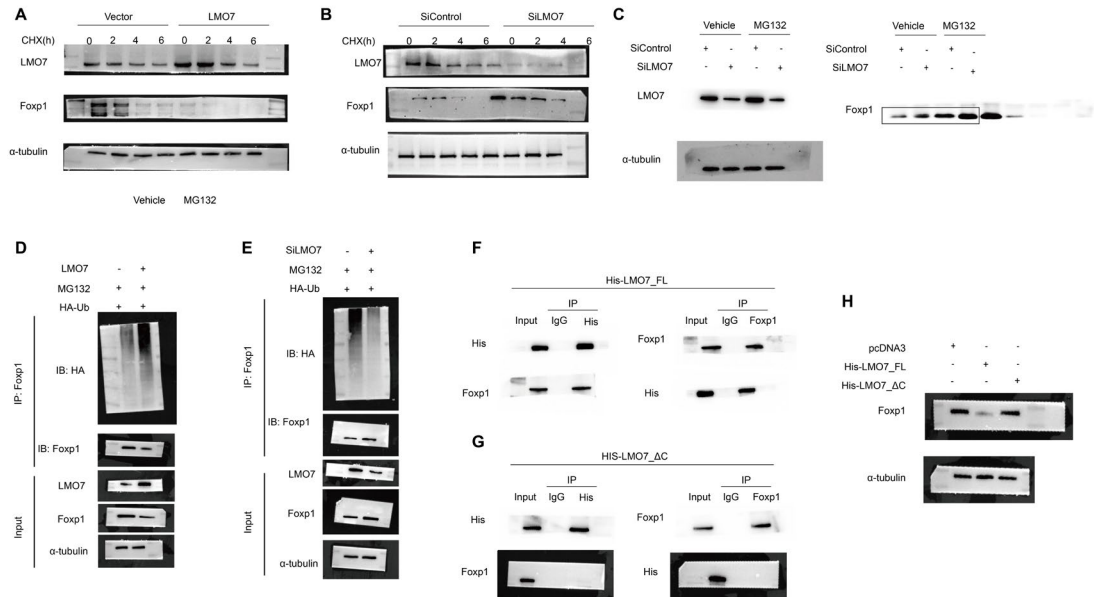

**Fig. S2**

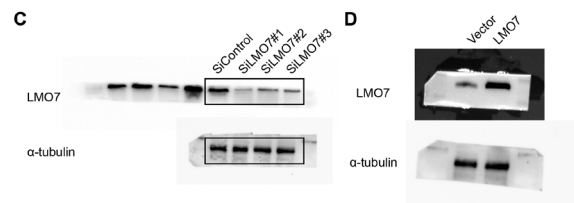

Supplement: Supplementary file 2 — Supplementary Materials-Original western blots [file 41418_2024_1358_MOESM2_ESM.pdf]
